# Variance in animal longevity: contributions of heterogeneity and stochasticity

**DOI:** 10.1007/s10144-018-0616-7

**Published:** 2018-05-28

**Authors:** Nienke Hartemink, Hal Caswell

**Affiliations:** Institute for Biodiversity and Ecosystem Dynamics, University of Amsterdam, P.O. Box 94248, 1090 GE Amsterdam, The Netherlands

**Keywords:** Age-stage classified model, Heterogeneity, Individual stochasticity, Mixture models, Variance in longevity, Weibull distribution

## Abstract

Variance in longevity among individuals may arise as an effect of heterogeneity (differences in mortality rates experienced at the same age or stage) or as an effect of individual stochasticity (the outcome of random demographic events during the life cycle). Decomposing the variance into components due to heterogeneity and stochasticity is crucial for evolutionary analyses.In this study, we analyze longevity from ten studies of invertebrates in the laboratory, and use the results to partition the variance in longevity into its components. To do so, we fit finite mixtures of Weibull survival functions to each data set by maximum likelihood, using the EM algorithm. We used the Bayesian Information Criterion to select the most well supported model. The results of the mixture analysis were used to construct an age × stage-classified matrix model, with heterogeneity groups as stages, from which we calculated the variance in longevity and its components. Almost all data sets revealed evidence of some degree of heterogeneity. The median contribution of unobserved heterogeneity to the total variance was 35%, with the remaining 65% due to stochasticity. The differences among groups in mean longevity were typically on the order of 30% of the overall life expectancy. There was considerable variation among data sets in both the magnitude of heterogeneity and the proportion of variance due to heterogeneity, but no clear patterns were apparent in relation to sex, taxon, or environmental conditions.

**Electronic supplementary material:**

The online version of this article (10.1007/s10144-018-0616-7) contains supplementary material, which is available to authorized users.

## Introduction

Individual variance in fitness components is central to evolutionary demography and ecology, since variation between individuals in their traits and the resulting consequences for fitness are the basis for natural selection. Longevity, or age at death, is such a fitness component that varies among individuals within a cohort or population. This variance in longevity may arise as a result of two different underlying causes: stochastic processes and heterogeneity between individuals. That is, even in a population without heterogeneity, in which all individuals experience identical age-specific mortality rates, death would be a probabilistic event, leading to variance in longevity among individuals. This source of variance is called *individual stochasticity* (Caswell 2009). On top of that, genuine heterogeneity in age-specific mortality risk among individuals can be a cause of variance. Such differences between individuals with respect to their mortality risk, especially unobserved differences, are often referred to as heterogeneity in individual frailty (Vaupel et al. 1979), where frailty is defined as proneness to mortality.

The impact of heterogeneity on demographic outcomes, eco-evolutionary processes, and population dynamics has been the topic of several studies (e.g., Kendall and Fox 2002; Robert et al. 2003; Kendall et al. 2011; Vindenes and Langangen 2015; Cam et al. 2016). However, the extent to which variance in fitness components can be accounted for by individual stochasticity is still open (Cam et al. 2016). This question is fundamental to evolutionary demography because the two sources of variance have very different implications. Although it can arise from many other causes, variance due to heterogeneity may have a genetic basis, and hence play a role in selection. Because variance due to individual stochasticity arises from individuals experiencing identical vital rates, by definition it cannot have a genetic basis. It may even slow down selection by obscuring genetic variance that does exist (Steiner and Tuljapurkar 2012). Automatically attributing observed variance in fitness components to heterogeneity overestimates the potential for selection.

The key to quantifying the relative contributions of heterogeneity and stochasticity is to construct a demographic model in which both factors operate, and partition the resulting variance into components due to each process. Such a method has been developed by using age$\times$stage-classified matrix population models (Caswell 2014; Hartemink et al. 2017). The primary state variable is age, and some aspect of heterogeneity (e.g., frailty) is included as a stage. When variance in, for example, longevity is calculated from such a matrix, it can be decomposed into a variance component between frailty classes, which is due to heterogeneity and a variance component within frailty classes, which is due to stochasticity. A first, crude attempt to explore this question in laboratory animal cohort data was made in Caswell (2014), but that analysis relied on previously published estimates of uncertain methodology, based on a restrictive mortality model, and with only an approximate variance decomposition. Here we present a more rigorous analysis.

We quantify the relative contribution of heterogeneity and stochasticity to the variance in longevity in a range of invertebrate laboratory animal studies, comprising 25 data sets on 9 species of nematodes and insects, totaling about 3.2 million individuals. Heterogeneity in mortality was captured by fitting finite mixtures of Weibull functions to data on individual ages at death, using the expectation–maximization (EM) algorithm. We used model selection criteria to choose the mixture model most well supported by the data, and constructed the corresponding matrix model to estimate the components of variance.

In this paper we address several questions: (1) is there evidence for unobserved heterogeneity in mortality of invertebrates under controlled conditions, (2) if so, how much, and how are individuals distributed among heterogeneity groups, and (3) how much of the variance in longevity is due to heterogeneity and how much to individual stochasticity. Because we are using data on a variety of species, on both sexes, and sometimes under different conditions, we will look for patterns related to these variables. But because our results are based on an arbitrary and non-random selection of data, constrained by sample size, rigorous comparative analyses are impossible.

We begin by describing the statistical estimation procedures and then develop the age $\times$ stage-classified matrix population model. Subsequent sections calculate and decompose the variance in longevity. We show the results in a series of tables and figures; detailed results for each data set are found in Electronic Supplementary Materials [ESM-1 and 2].

## Materials and methods

### Finite mixture models for survival

We described heterogeneity by a finite mixture model, in which a discrete number *g* of groups are defined, each group having its own mortality parameters, and with group *i* comprising a proportion $\pi _i$ of the cohort at the initial age. The mortality parameters of each group and the mixing distribution $\varvec{\pi }$ are estimated by maximum likelihood from data on the observed distribution of age at death.

Such finite mixture models are widely used in statistics (e.g., McLachlan and Peel 2004; Frühwirth-Schnatter 2006). They have long been applied in survival analysis as an alternative to continuous frailty models (Farewell 1982; Heckman and Singer 1982; McLachlan and McGiffin 1994; Erişoğlu et al. 2012) such as the Gamma–Gompertz model (Vaupel and Carey 1993). Bijwaard (2014) and Putter and van Houwelingen (2015) have explored finite mixtures in multistate models.

We used the Weibull distribution to model survival. This distribution is more flexible than the Gompertz model, because it permits increasing, decreasing, or constant hazard rates, thus incorporating the type I, II, and III survivorship curves familiar in ecology (Pinder et al. 1978). The Weibull distribution has appealing biological interpretations as the time to failure of a system that relies on the continued operation of a large number of processes and fails when any one of them does so (e.g., Horvath 1968), or as the result of accumulation of damage beyond a certain threshold (Rinne 2008). The Weibull hazard function is:

1 $$\begin{aligned} \mu (x|\lambda ,k)= \frac{k}{\lambda } \left( \frac{x}{\lambda }\right) ^{k-1} \end{aligned}$$

where *x* is age, $\mu (x)$ is the mortality hazard, $\lambda$ is a scale parameter, and *k* is a shape parameter. In medical statistics, alternative parameterizations are sometimes used (e.g., Mills 2011), in which the shape parameter is the same as above, but the scale parameter is $\lambda ^{(-k)}$ in our parametrization. In Matlab, the model is specified using *a* for the scale parameter and *b* for the shape parameter. If $k\le 1$, the hazard increases with time. If $k>1$, the hazard decreases over time. If $k = 1$, the hazard is constant and the model reduces to an exponential model. The probability density function of age at death for the Weibull distribution is:

2 $$\begin{aligned} f(x|\lambda ,k)= \frac{k}{\lambda }\left( \frac{x}{\lambda }\right) ^{k-1} \exp \left( -\left( \frac{x}{\lambda } \right) ^{k} \right). \end{aligned}$$

#### Maximum likelihood estimation

The mixture models were fit to the data using maximum likelihood. The estimation of mixture models is, in general, difficult, but the expectation–maximization (EM) algorithm (Dempster et al. 1977) has made it widely possible. The EM algorithm is an iterative procedure that alternates between an expectation (E) step and a maximization (M) step, until the estimates converge (for details, see McLachlan and Krishnan (2007)). Conceptually, it treats group membership as missing data. In the E step, the expected value of the unknown group membership is calculated for each individual, given the survival parameters in each group. The M step then finds parameters that maximize the likelihood, given the expected group memberships of each individual. Then the expectation step is repeated with the new parameters, and so on. See McLachlan and McGiffin (1994) for a general discussion of the EM algorithm in relation to survival analysis. We programmed our analysis following the application of the EM algorithm to survival data by Mohammed et al. (2013). The approach has been shown by simulation studies to be capable of distinguishing mixtures of Weibull (and other) distributions (e.g., Erişoğlu et al. 2011, 2012; Mohammed et al. 2015).

To help ensure convergence to global rather than local maxima of the likelihood function, we sampled at least ten initial values for the parameters. We selected the best estimate of the number of groups; following the suggestion of Frühwirth-Schnatter (2006) we used the minimum Bayesian Information Criterion (BIC) as our criterion. This lessens the risk of overfitting the number of heterogeneity groups. Runs that resulted in values of *k* greater than 10 were excluded, because values of $k>10$ produce extremely narrow distributions of age at death, indicating that the model is trying to fit a few data points instead of a general distribution.

### A matrix model including heterogeneity

*Notation* Matrices are denoted by upper-case boldface letters (e.g., **U**), and vectors by lower-case boldface letters (e.g., **n**). Block-diagonal matrices are denoted by blackboard font (e.g., $\mathbb {U}$). A tilde is used to distinguish matrices and vectors associated with the full age$\times$stage-classified model, e.g., by $\tilde{\mathbf{U }}$, $\tilde{\mathbf{n }}$; these matrices are block-structured and contain entries for all combinations of age classes and heterogeneity groups. The identity matrix of order *s* is denoted **I**$_s$, and $\varvec{1}_s$ is a $s \times 1$ vector of ones. The unit vector **e**$_i$ is a vector with a 1 in the *i*th entry and zeros elsewhere. The symbol $\circ$ denotes the Hadamard, or element-by-element product; the symbol $\otimes$ denotes the Kronecker product. The transpose of the matrix **X** is **X**$^{{\tiny \mathsf T}}$. The matrix **K** is the vec-permutation matrix (Henderson and Searle 1981).

The estimated number of groups (which we will denote by *g*), the proportion of individuals in each group (described by the mixing distribution vector $\varvec{\pi }$) and the Weibull parameters $\lambda$ and *k* for each group serve as input for our age$\times$stage-classified matrix model. The stages are groups, each with its own mortality function, where the age-specific hazard is specified by the estimated Weibull parameters for that particular group.

The state of an individual is given by its age and its heterogeneity group. To include both these variables, we use an age$\times$stage-classified matrix model, in which individuals are jointly classified by age and stage (Caswell 2012). In this case, stages are heterogeneity groups. Each group has an age-dependent mortality schedule specified by its Weibull parameters. In general, an age $\times$ stage-classified model describes both progression through age classes and transitions among stages (Caswell 2012). However, the heterogeneity groups here are fixed, so we need not include transitions among them (but see Hartemink et al. (2017), Caswell (2014) and Caswell et al. (2018) for more details on how to include such transitions).

Let $\omega$ be the number of age classes and *g* be the number of heterogeneity groups. The population vector $\tilde{\mathbf{n}}$ is

3 $$\begin{aligned} \tilde{\mathbf{n}} = \left( \begin{array}{ll} n_{11} \\ \vdots \\ n_{1g} \\ \hline \vdots \\ \hline n_{ \omega 1} \\ \vdots \\ n_{ \omega g} \end{array}\right) \end{aligned}$$

where the *j*th block of entries in $\tilde{\mathbf{n}}$ is a sub-vector describing the abundance of the *g* stage classes within age class *j*.

For each group *i*, define a survival matrix $\mathbf{U}_i$ of dimension $\omega \times \omega$ that contains age-specific survival probabilities on the first subdiagonal and zeros elsewhere,

4 $$\begin{aligned} \mathbf{U}_i = \left( \begin{array}{llll} 0 &{} 0 &{} \cdots &{} 0\\ e^{-\mu _i(0)} &{} 0 &{} \cdots &{} 0\\ \vdots &{} \ddots &{} &{} \vdots \\ 0 &{} \cdots &{} e^{-\mu _i(\omega -1)} &{} 0 \end{array}\right) \end{aligned}$$

where $\mu _i(x)$ is the mortality rate at age *x*, given by Eq. 1, for group *i*. Create a block-diagonal matrix $\mathbb {U}$ (of dimension $\omega g \times \omega g$) by placing the $\varvec{U}_i$ on the diagonal,

5 $$\begin{aligned} \mathbb {U} = \left( \begin{array}{lll} \mathbf{U}_1 &{} \cdots &{} 0 \\ \vdots &{} \ddots &{} \vdots \\ 0 &{} \cdots &{} \mathbf{U}_g \end{array}\right) . \end{aligned}$$

The joint age $\times$ stage composition of the cohort at time *x* is projected as

6 $$\begin{aligned} \tilde{\mathbf{n}}(x+1) = \tilde{\mathbf{U}} \tilde{\mathbf{n}}(x) \end{aligned}$$

where the projection matrix is

7 $$\begin{aligned} \tilde{\mathbf{U}} =\mathbf{K}^{{\tiny \mathsf T}}\mathbb {U} \mathbf{K} \end{aligned}$$

with ${\varvec{K}} = {\varvec{K}}_{g,\omega }$ the vec-permutation matrix (Henderson and Searle 1981; Hunter and Caswell 2005; Caswell 2012), which rearranges the population vector to permit multiplication by the block diagonal matrix.

### Calculating longevity

The matrix $\tilde{\mathbf{U}}$ is the transient matrix of an absorbing Markov chain, with death as an absorbing state (e.g., Caswell 2001, 2009, 2014). The fundamental matrix of this chain (of dimension $\omega g \times \omega g$) is

8 $$\begin{aligned} \tilde{\mathbf{N}} = \left( \mathbf{I}_{\omega g} - \tilde{\mathbf{U}} \right) ^{-1} \end{aligned}$$

where $\mathbf{I}_{\omega g}$ is an identity matrix. The (*x*, *y*) entry of $\tilde{\mathbf{N}}$ is the expected number of visits to state *y* by an individual in state *x*, where state refers to the specific combination of age and stage.

The statistics of longevity are calculated from $\tilde{\mathbf{N}}$ (e.g., Caswell 2009). The vectors of first and second moments of longevity, are given by

9 $$\begin{aligned} \tilde{\varvec{\eta }}_1= & {} \left( \varvec{1}_\omega ^{{\tiny \mathsf T}}\tilde{\mathbf{N}} \right) ^{{\tiny \mathsf T}}\qquad g \omega \times 1\end{aligned}$$

10 $$\begin{aligned} \tilde{\varvec{\eta }}_2= & {} \left[ \tilde{\varvec{\eta }}_1^{{\tiny \mathsf T}}\left( 2 \tilde{\mathbf{N}} - \mathbf{I}_{\omega g} \right) \right] ^{{\tiny \mathsf T}}\qquad g \omega \times 1 \end{aligned}$$

These vectors contain the moments of the longevity of all $g \omega$ age$\times$stage combinations. The vector of mean life expectancies (mean longevities) of each age$\times$stage combination is $\tilde{\varvec{\eta }}_1$. The vector of variances in longevity is

11 $$\begin{aligned} V(\tilde{\varvec{\eta }})= & {} \tilde{\varvec{\eta }}_2 - \tilde{\varvec{\eta }}_1 \circ \tilde{\varvec{\eta }}_1 \qquad g \omega \times 1 \end{aligned}$$

We are interested in the remaining longevity from the start of the cohort (age class 1), so we extract the mean and variance of longevity at age 1 from the full vectors. Define a vector $\varvec{\eta }_{\rm groups}$, of dimension $g \times 1$, that contains the longevity, at age 1, of individuals in each of the heterogeneity groups. The mean and variance of $\varvec{\eta }_{\rm {groups}}$ are

12 $$\begin{aligned} E(\varvec{\eta }_{\rm groups})= & {} \left( \mathbf{e} _1^{{\tiny \mathsf T}}\otimes \mathbf{I}_g \right) \tilde{\varvec{\eta }}_1 \qquad g \times 1 \end{aligned}$$

13 $$\begin{aligned} V(\varvec{\eta }_{\rm groups})= & {} \left( \mathbf{e}_1^{{\tiny \mathsf T}}\otimes \mathbf{I}_g \right) V(\tilde{\varvec{\eta }}) \qquad g \times 1 \end{aligned}$$

where **e**$_1$ is a vector of length $\omega$ with a 1 in the first entry and zeros elsewhere and **I**$_g$ is an identity matrix of size *g*.

### Variance decomposition: heterogeneity and stochasticity

The first age class is a mixture of individuals with a mixing distribution $\varvec{\pi }$ (which is a vector giving the fractions of the population in each group); $\varvec{\pi }$ is estimated by the EM algorithm. The variance in longevity of age class 1, considered as a mixture of groups, is

14 $$\begin{aligned} V(\eta )=\; & {} E_{\pi } \left[ V \left( \varvec{\eta }_{\rm groups} \right) \right] + V_\pi \left[ E \left( \varvec{\eta }_{\rm groups} \right) \right] \end{aligned}$$

15 $$\begin{aligned} \quad \;\;\; =\; V_{\rm within} + V_{\rm between} \qquad 1 \times 1 . \end{aligned}$$

The first term is the within-group variance; it is the weighted mean of the group variances in the vector V($\varvec{\eta }_{\rm groups}$), as given by Eq. 13,

16 $$\begin{aligned} V_{\rm within}= & {} \varvec{\pi }^{{\tiny \mathsf T}}V(\varvec{\eta }_{\rm groups}) \end{aligned}$$

17 $$\begin{aligned} \qquad \, \; = \left( \mathbf{e}_1^{{\tiny \mathsf T}}\otimes \varvec{\pi }^{{\tiny \mathsf T}}\right) V(\tilde{\varvec{\eta }}) \qquad 1 \times 1 . \end{aligned}$$

The second term is the between-group variance; it is the weighted variance of the group means in the vector E($\varvec{\eta }_{\rm groups}$), as given by Eq. 12,

18 $$\begin{aligned} V_{\rm between}=\; & {} \varvec{\pi }^{{\tiny \mathsf T}}\left[ E(\varvec{\eta }_{\rm groups}) \circ E(\varvec{\eta }_{\rm groups}) \right] \nonumber \\&- \left[ \varvec{\pi }^{{\tiny \mathsf T}}E(\varvec{\eta }_{\rm groups} ) \right] ^2 \end{aligned}$$

19 $$\begin{aligned} \qquad \quad = & \, \varvec{\pi }^{{\tiny \mathsf T}}\left[ \left( \mathbf{e}_1^{{\tiny \mathsf T}}\otimes \mathbf{I}_g \right) \tilde{\varvec{\eta }}_1 \circ \left( \mathbf{e}_1^{{\tiny \mathsf T}}\otimes \mathbf{I}_g \right) \tilde{\varvec{\eta }}_1\right] \nonumber \\&- \left[ \left( \mathbf{e}_1^{{\tiny \mathsf T}}\otimes \varvec{\pi }^{{\tiny \mathsf T}}\right) \tilde{\varvec{\eta }}_1 \right] ^2 \qquad 1 \times 1 . \end{aligned}$$

The within-group variance component measures the variance due to individual stochasticity among individuals experiencing the same group-specific mortality schedule. The between-group component measures the variance due to the differences in the mortality schedules among the groups. In the absence of heterogeneity, the variance among group means would be zero and all variance would be due to stochasticity. In the absence of stochasticity, all the group variances would be zero and all variance would be due to heterogeneity. Thus the between-group variance, as a fraction of the total, is a measure of the contribution of heterogeneity to variance in longevity.

### The magnitude of heterogeneity

We measured the amount or magnitude of heterogeneity in two ways. First, we consider the concentration of individuals within groups. Heterogeneity is less if individuals are concentrated in one or a few groups than if they are spread out among groups in relatively equal proportions. We measure this concentration by the entropy of the mixing distribution

20 $$\begin{aligned} H = - \sum _{i=1}^g \pi _i \log \pi _i \end{aligned}$$

which has its maximum value $H=\log g$ when all the $\pi _i$ are equal. Because the entropy is affected by the number of groups as well as the distribution of individuals among the groups, we scale it relative to its maximum to obtain the evenness,

21 $$\begin{aligned} J = \frac{H}{\log g} \end{aligned}$$

which ranges from 0 (in the limit as all individuals are concentrated in one group) to 1 (individuals equally distributed among groups).

Second, we consider the magnitude of differences among the groups. Heterogeneity is less when the differences among groups are small than when they are large. We measured the magnitude of the differences among groups by the between-group standard deviation; i.e., the square root of the between-group variance (Eq. 18). In order to compare species with different life expectancies, we scaled the standard deviation by the overall life expectancy.

### Data

We obtained individual survival data from the literature or from the DATLife database (DATLife 2017), choosing studies with large sample sizes to permit rigorous statistical analysis. All data were obtained from laboratory studies under constant (to the best efforts of the original investigators) conditions. In the end, we analyzed 25 data sets on nine species of invertebrates (one nematode and 8 insects). Some of the data were additionally broken down by sex or genetic strains. The sample sizes and characteristics of the data are shown in Table 1. More detailed information on species, sources of data, and experimental conditions is given in Appendix 1.

**Table 1:** Characteristics of the data sets analyzed in the paper, showing sample size (*N*), life expectancy (LE) in days, the observed variance in age at death, and the maximum observed life span in days

Species strain/sex	Raw mortality data			
	*N*	LE	Variance	Max. life span
*C. elegans*				
N2	1000	14.32	26.20	33
CLK-1	800	18.24	106.48	55
DAF-2	800	30.19	224.64	62
All	2600	20.40	156.93	62
Human louse				
Females	400	17.08	84.31	46
Males	400	17.17	63.59	44
Both sexes	800	17.12	73.36	46
Housefly				
Females	3875	28.74	146.14	65
Males	4627	16.93	45.60	58
Both sexes	8502	22.22	123.01	65
*Anastrepha ludens*				
Females	363,971	28.54	198.86	163
Males	487,128	30.31	196.53	155
Both sexes	851,099	29.56	198.29	163
*A. obliqua*				
Females	134,807	17.91	147.07	84
Males	162,280	15.43	100.67	67
Both sexes	297,087	16.55	123.24	84
*A. serpentina*				
Females	169,031	17.96	96.72	90
Males	172,283	18.57	113.74	77
Both sexes	341,314	18.27	105.40	90
*D. longicaudata*				
Females	14,184	8.60	38.25	70
Males	13,358	7.97	30.79	64
Both sexes	27,542	8.29	34.73	70
Drosophila				
Long-winged females	5426	38.00	406.93	97
Long-winged males	4568	40.40	433.58	95
Short-winged females	906	15.26	112.16	46
Short-winged males	854	13.67	79.91	51
All	11,754	35.42	449.17	97
Medfly diet study				
Females sugar only	101,362	12.89	58.40	97
Males sugar only	106,662	14.23	56.18	79
Females sugar+protein	99,312	14.35	64.51	133
Males sugar+protein	108,953	14.18	51.98	64
All	416,289	13.92	57.95	133
Million medfly study				
Females	605,528	19.58	87.56	171
Males	598,118	22.13	80.45	164
Both sexes	1,203,646	20.84	85.65	171

## Results

From each data set, we obtained the estimated number *g* of groups (selected by minimizing BIC), the Weibull parameters for each group, and the proportions of each group in the initial cohort. From these, we constructed the age$\times$stage-classified matrix model and partitioned the variance in longevity into components due to heterogeneity and individual stochasticity, and obtained the proportion of the variance due to heterogeneity. The resulting values are shown in Table 2. In Electronic Supplementary Material [ESM-1], we provide the complete set of all estimates, not just those for the model selected by minimizing BIC.

### Detailed results for *C. elegans*

**Fig. 1:**
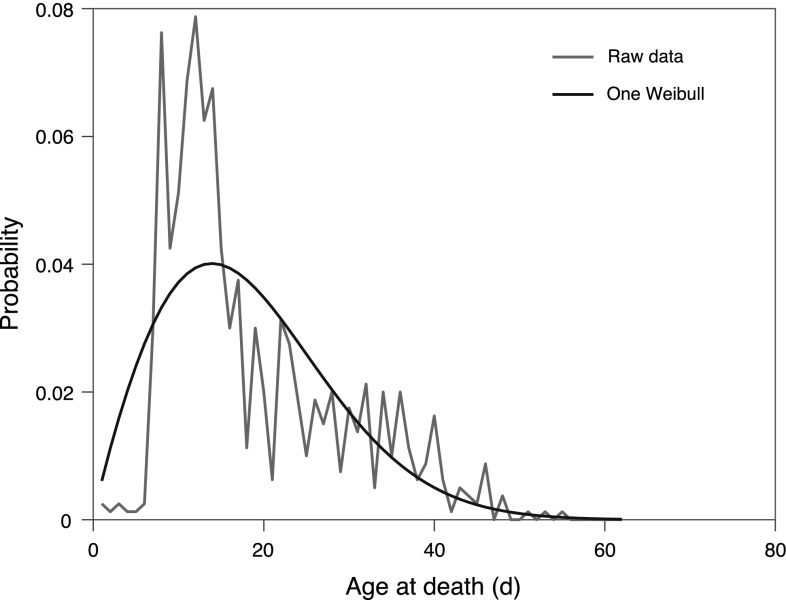
Age-at-death of *C. elegans* strain CLK-1: raw data (red) and modelled by a single Weibull function (black)

To clarify the analyses and as an example of the procedure, we present here the detailed results for a cohort of 800 individuals of the CLK-1 strain of the nematode *C. elegans* from an experiment described in Chen et al. (2007). Fitting a single Weibull function to the age-at-death data of this strain yields an estimate of $\lambda$ = 20.7 and *k* = 1.9. From Fig. 1, it is clear that a single Weibull function does not provide a good fit to the data.

The results of fitting mixtures of two, three, four, or five Weibull functions are shown in Table 3 (models with mixtures of six, seven, or eight Weibull functions either did not converge or produced results with $k>10$).

**Fig. 2:**
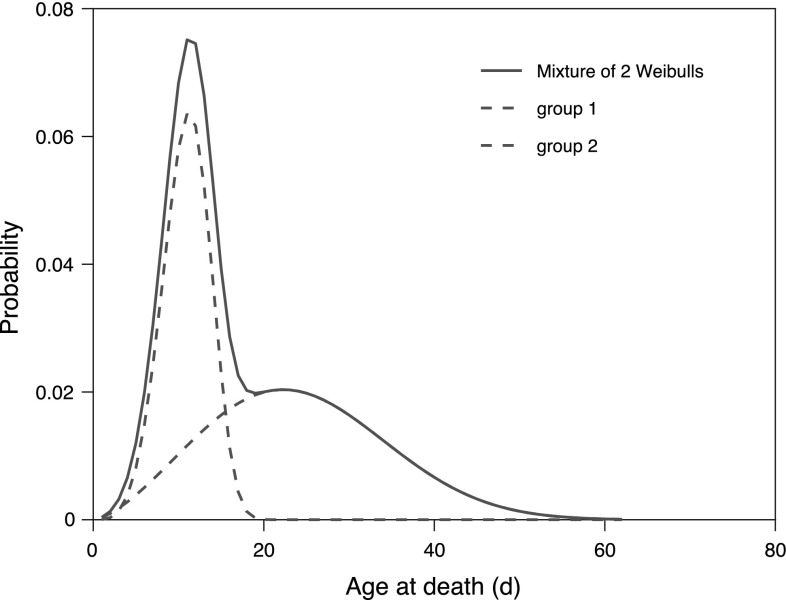
*C. elegans* CLK-1: fitted Weibull functions for age-at-death for the weighted mixture and for the two groups

**Fig. 3:**
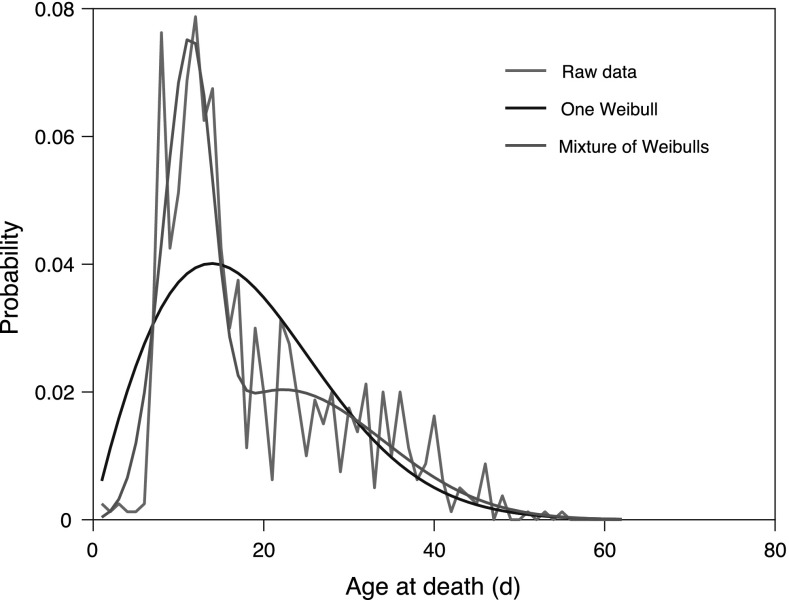
Age-at-death of *C. elegans* strain CLK-1: raw data (red), modelled as single Weibull function (black) and modelled as a mixture of two Weibull functions (blue)

Based on the BIC values, we conclude that a mixture of two Weibull functions is the model most well supported by these data. The first group, comprising 45% of the individuals, is characterized by Weibull parameters $\lambda = 11.9$ and $k = 4.5$. The other group, comprising the remaining 55% of the individuals, has Weibull parameters $\lambda =27.3$ and $k=2.5$. These two functions, scaled by their mixing proportions, and their mixture are shown in Fig. 2. The first group is characterized by a shorter and less variable longevity (modal age at death 11 days), the second group by a longer and more variable life span (modal age at death 22 days). When comparing the raw data, a single Weibull, and a mixture of two Weibull functions (Fig. 3), it is clear that the mixture model provides the better fit.

These estimated parameters are used as input for our age$\times$stage-classified matrix model. The number of groups is $g = 2$ in this case. We used 200 age classes ($\omega =200$). The mixing distribution $$\varvec{\pi }= \left( \begin{array}{ll} 0.45&0.55 \end{array}\right)^{{\tiny \mathsf T}}$$; this nearly equal division into two groups yields an evenness of 0.99. The age-specific mortality hazards for the two groups are

22 $$\begin{aligned} \mu _1(x)= & {} \frac{4.5}{11.9} \left( \frac{x}{11.9}\right) ^{4.5-1} \end{aligned}$$

23 $$\begin{aligned} \mu _2(x)= & {} \frac{2.5}{27.3} \left( \frac{x}{27.3}\right) ^{2.5-1} \end{aligned}$$

These hazard functions determine the age-specific survival probabilities in the $\mathbf{U}_i$ matrices in Eq. 4. From this, the block-diagonal matrix $\mathbb {U}$ and the projection matrix $\tilde{\mathbf{U}}$ are derived using Eqs. 5 and 7. The mean longevity in the heterogeneous cohort is 19.2 d. The variance is 106 d$^2$; of this variance, 41.4% is due to heterogeneity between the groups, and the remaining 58.6% is due to individual stochasticity. The among-group standard deviation is 35% of the mean longevity.

The Matlab scripts for estimating the parameters and BIC values for each model using the EM algorithm and for calculating longevity statistics and decomposing the variance, can be found in the Electronic Supplementary Material [ESM-3].

### Results: species comparison

Table 2 shows the results for the number of heterogeneity groups identified, the evenness of the distribution of individuals among groups, the magnitude of the differences among groups, and the fraction of variance due to heterogeneity. In only four cases (both sexes of the human louse, the N2 strain of *C. elegans*, and males of the short-winged strain of *Drosophila*) did we fail to find evidence of heterogeneity. In the other cases, populations were quite evenly distributed among groups, with a median evenness (*J*) of 0.75 (interquartile range 0.41–0.89).

The magnitude of the heterogeneity (the among-group standard deviation) had a median value of 28% of life expectancy (interquartile range 24–34%). Heterogeneity accounts for a substantial but not overwhelming fraction of the variance in longevity. The median contribution of heterogeneity is 35% (interquartile range 23–44%). The highest contributions are 75% in *Anastrepha obliqua* females and 65% in the DAF-2 strain of *C. elegans*.

**Table 2:** Results of mixture model analysis

Species strain/sex	Best fitting mixture of Weibull functions								
	*g*	LE	Variance	Within	Between	Entropy	Evenness	Ratio	% of variance
*C. elegans*									
N2	1	15.33	26.82	26.82	0.00	0.00	–	0.0	0.0
CLK-1	2	19.22	106.45	62.38	44.07	0.69	0.99	0.35	41.4
DAF-2	2	31.21	223.75	78.90	144.85	0.61	0.88	0.39	64.7
All	4	21.42	156.78	46.83	109.95	1.11	0.80	0.39	70.1
Human louse									
Females	1	18.06	85.28	85.28	0.00	0.00	–	0.0	0.0
Males	1	18.14	62.76	62.76	0.00	0.00	–	0.0	0.0
Both sexes	1	18.10	74.27	74.27	0.00	0.00	–	0.0	0.0
Housefly									
Females	2	29.73	146.83	91.01	55.83	0.44	0.64	0.25	38.0
Males	4	17.94	45.84	32.95	12.89	1.10	0.79	0.20	28.1
Both sexes	4	23.30	125.81	70.11	55.70	0.97	0.70	0.32	44.3
*Anastrepha ludens*									
Females	6	29.55	198.62	126.76	71.86	1.44	0.80	0.29	36.2
Males	5	31.32	196.35	158.19	38.16	0.81	0.50	0.20	19.4
Both sexes	5	30.56	198.08	160.74	37.34	1.04	0.65	0.20	18.9
*A. obliqua*									
Females	6	18.93	146.65	37.29	109.35	1.72	0.96	0.55	74.6
Males	6	16.45	100.38	32.12	68.26	1.67	0.93	0.50	68.0
Both sexes	6	17.59	122.77	69.77	53.01	1.28	0.71	0.41	43.2
*A. serpentina*									
Females	5	18.97	96.47	78.43	18.04	0.80	0.50	0.22	18.7
Males	5	19.59	113.40	82.87	30.53	1.04	0.65	0.28	26.9
Both sexes	5	19.29	105.07	82.09	22.98	1.14	0.71	0.25	21.9
*D. longicaudata*									
Females	4	9.64	38.07	27.70	10.37	1.31	0.95	0.33	27.2
Males	4	9.05	30.45	25.77	4.69	0.71	0.51	0.24	15.4
Both sexes	4	9.36	34.36	28.95	5.41	0.85	0.61	0.25	15.7
Drosophila									
Long-winged females	2	39.06	403.70	240.11	163.59	0.57	0.82	0.33	40.5
Long-winged males	2	41.43	431.64	239.23	192.41	0.52	0.76	0.33	44.6
Short-winged females	2	16.28	111.82	50.83	60.99	0.65	0.93	0.48	54.5
Short-winged males	1	14.76	77.23	77.23	0.00	0.00	–	0.00	0.0
All	2	36.46	446.91	213.69	233.22	0.64	0.92	0.42	52.2
Medfly diet study									
Females sugar only	4	13.94	58.10	44.85	13.25	1.24	0.89	0.26	22.8
Males sugar only	4	15.27	55.88	50.47	5.41	1.05	0.75	0.15	9.7
Females sugar + protein	4	15.40	64.04	44.80	19.23	1.31	0.94	0.28	30.0
Males sugar + protein	4	15.23	51.60	38.72	12.88	1.30	0.94	0.24	25.0
All	4	14.97	57.55	44.06	13.49	1.38	1.00	0.25	23.4
Million medfly study									
Females	6	20.58	87.58	53.13	34.44	1.37	0.76	0.29	39.3
Males	8	23.14	80.47	53.00	27.47	1.71	0.82	0.23	34.1
Both sexes	8	21.85	85.66	50.10	35.56	1.56	0.75	0.27	41.5

The number of groups (*g*) that results in the lowest BIC, the life expectancy (LE), the total variance in longevity, the within- and between-class variance, the entropy (*H*) and evenness (*J*) indices, the ratio between the square root of the between variance and the life expectancy, and the percentage of variance due to heterogeneity

**Table 3:** Best fits for models using mixtures of up to five Weibull functions for the CLK-1 strain of *C. elegans*

*g*	Group 1			Group 2			Group 3			Group 4			Group 5			$\Delta$BIC
	$\lambda$	*k*	%	$\lambda$	*k*	%	$\lambda$	*k*	%	$\lambda$	*k*	%	$\lambda$	*k*	%	
1	20.7	1.9	100													167.23
2	11.9	4.5	45.0	27.3	2.5	55.0										0
3	12.0	4.3	49.2	0.8	1.9	0.3	28.6	2.8	50.5							8.51
4	19.6	4.6	15.7	1.2	1.5	0.4	32.8	3.7	34.0	11.8	4.4	0.50				20.80
5	37.1	4.5	14.8	20.7	4.1	25.2	11.8	4.4	49.5	33.5	7.3	10.0	1.2	1.5	0.4	40.12

For each model the number of groups (*g*), the estimated Weibull parameters $\lambda$ and *k*, the percentage of the population made up by each group are shown. Also shown is $\Delta$BIC, the difference from the minimum value of BIC, which corresponds to the best model

## Discussion

We set out to address three questions: is there evidence for heterogeneity, if so how much, and what fraction of the variance in longevity is due to heterogeneity, and what fraction to stochasticity. We found statistical support for heterogeneity in 31 out of 35 cases. In only four data sets (the N2 strain of *C. elegans*, both sexes of the human louse, and males of the short-winged strain of *Drosophila*) was a homogeneous model, with only a single group, the best supported. These were among the smallest data sets in our studies (1,000 individuals for *C. elegans*, 400 of each sex for the louse, and 854 for the short-winged *Drosophila*). It would not be surprising if heterogeneity is more difficult to detect in small samples. Had these experiments been performed with more individuals, multiple groups might have been identified.

For all other data sets, our analysis identified from 2 to 6 heterogeneity groups. Generalizing from these results, we can say that individuals are relatively evenly spread out among the groups, with an evenness of about 75% of its maximum. The differences among groups in life expectancy are about 28% of overall life expectancy.

Partially because of its evolutionary implications, much of the interest in unobserved heterogeneity in fitness components focuses on accounting for variance (e.g., Caswell 2011, 2014; Steiner and Tuljapurkar 2012; Vindenes and Langangen 2015; Cam et al. 2016; Hartemink et al. 2017; van Daalen and Caswell 2017; Jenouvrier et al. 2018). In this case, we found that heterogeneity could typically account for less than half of the variance in longevity (35%, with interquartile range 23–44%).

We found substantial differences among species in the number of groups distinguished and in the proportion of the variance attributable to heterogeneity. However, we found no clear patterns involving differences between sexes, treatments, or strains. Application of this approach to other experimental studies, in which large numbers of individuals are exposed to different treatments, would be valuable.

The very large datasets (the *Anastrepha* species and the Million Medfly experiment) seem to reveal higher numbers of groups; this may reflect an increased ability to detect heterogeneity with large sample sizes. There is no correlation between the number of groups and the fraction of variance due to heterogeneity; both high and low numbers of groups can result in high fraction of variance due to heterogeneity. For example, the fraction of variance due to heterogeneity was high with only two groups (e.g., 65% in the DAF-2 strain of *C. elegans*) and with six groups (e.g., 75% in female *A. obliqua*).

Note that the value of the estimated longevity is in all cases approximately one unit higher than the longevity in the raw data, this is caused by the matrix model assumption of one remaining unit of life expectancy, even at the time of death. If we subtract this unit here, the estimated longevities match the raw data very well. The estimated variance also closely matches the variance as calculated from the raw data.

There exist a few studies to which this one can be compared. Heterogeneity makes a larger contribution to variance in longevity in this study than was previously found for humans in an analysis of cohort and period mortality patterns, over many years, for populations of Sweden, France, and Italy (Hartemink et al. 2017). In that analysis, heterogeneity accounted for less than 10%, and usually less than 5%, of the variance in longevity. It was based on a continuous heterogeneity model, in which a Gamma-distributed frailty term, acting as a proportional hazard on mortality, was applied to a Gompertz–Makeham mortality model (Missov 2013; Missov and Lenart 2013). The Gompertz–Makeham model is applicable to human populations only after the age of 30–40 years, so the human data corresponded to a later “adult” age than is the case for the invertebrate species studied here.

Caswell (2014) made a brief exploration of laboratory data on six species of invertebrates using Gamma–Gompertz parameters reported by Horiuchi (2003). A crude, approximate, variance decomposition found about 60% of the variance in longevity to be due to heterogeneity. However, because it is now known that neglecting the Makeham mortality term can bias estimates of the Gompertz parameters (Missov and Németh 2016), and because of the ad hoc variance decomposition used in Caswell (2014), we view these results as suggestive but not reliable.

In a recent field study of the Southern fulmar (*Fulmarus glacialoides*), Jenouvrier et al. (2018) used multievent capture-recapture analysis to identify three unobserved heterogeneity groups, where the groups were allowed to differ in any of the transition probabilities in a stage-classified matrix population model. They decomposed variance in longevity, age at first breeding, and lifetime reproductive output into contributions from heterogeneity and stochasticity. Heterogeneity accounted for only 5.9% of the variance in longevity, 3.7% of the variance in age at first breeding, and 22% of the variance in lifetime reproductive output.

Insects undergo metamorphosis before reaching the adult stage, and in some laboratory conditions (e.g., *Drosophila* culture bottles), crowded larval conditions may create heterogeneity through competition, with some individuals completing the larval stage, but less well equipped for the adult stage, leading to ‘early failure’^1^. Such early deaths would probably form a group with very low mean longevity, and this would contribute substantially to the variance, and also increase the contribution of heterogeneity to the variance in longevity.

The finite mixture approach to estimating heterogeneity has advantages over other frailty analyses. It does not require an assumption of a parametric mixing distribution (e.g., the Gamma distribution), nor does it require an assumption of how the heterogeneity acts. In the Gamma–Gompertz–Makeham model, for instance, heterogeneity acts as a proportional factor multiplying a baseline hazard (Vaupel and Missov 2014). In our approach, the number of groups, the distribution of individuals among the groups, and the scale and shape parameters of each of the Weibull functions are estimated without restriction.

An important direction for future research is the incorporation of dynamic heterogeneity, in which group membership is not fixed over the life of the individual. It will not be easy to estimate unobserved heterogeneity in these models (e.g., Putter and van Houwelingen 2015). However, when the heterogeneity can be observed or measured, dynamic transitions may be incorporated following the methods of multistate event history analysis (Willekens 2014).

Demographic components of fitness (longevity, lifetime reproductive output, age at first breeding, etc.) are important components of evolutionary demography. The variance in these components, if due to genetic heterogeneity, would provide material for natural selection. The results presented here, and those for recent human populations (Hartemink et al. 2017) and one long-lived seabird (Jenouvrier et al. 2018), suggest that for longevity, most of the variance is due to individual stochasticity. An understanding of the factors that influence this proportion, and the patterns shown by other taxa, are important research questions.

### Electronic supplementary material

Below is the link to the electronic supplementary material.

Supplementary material 1 (zip 136 KB)

Supplementary material 2 (pdf 415 KB)

Supplementary material 3 (zip 10 KB)

## Appendix: Species and data

This appendix gives details of the species and experimental sources for the data analyzed. Detailed comparisons of the raw data and the estimated Weibull functions are given for all data sets in ESM-2.

1. *Caenorhabditis elegans* This is a free-living nematode species and an often-used model organism. Mortality data on *C. elegans* was obtained from a experiment by Chen et al. (2007). In this experiment, the longevity of a standard wild-type strain (N2) and two long-lived mutant strains (CLK-1 and DAF-2) were reported. Daily survival data on 1000 individuals of the N2 strain and 800 individuals of each of the mutant strains were available.
2. *Pediculus humanus* (Human louse) The human louse (*Pediculus humanus* L.) is a small, wingless insect. Human lice are obligate parasites of humans, that is, they normally feed exclusively on human blood, but they can be reared successfully in the lab on rabbits. The life cycle consists of the egg, three larval instars and the adult stage. Data on adult survival of head lice were obtained from a study by Evans and Smith (1952), in which 800 freshly emerged adults (400 males and 400 females) were kept in mixed colonies. The mean duration of life was 17.6 for both males and females; there was no significant difference between the two sexes.
3. *Musca domestica* (Common House fly) The common house fly (*Musca domestica* L.) is a well-known fly species (Insecta:Diptera:Muscidae). We use data on adult life span from life tables for 4,627 males and 3,875 females published in Rockstein and Lieberman (1959). The data originally came from 2 separate studies, but no difference in mean length of life was found between these two datasets, so we treated it as one dataset. The house flies belonged to the strain NAIDM and the flies were inbred for about 200 generations.
4. *Anastrepha ludens* Also known as the Mexican fruit fly (Insecta:Diptera:Tephritoidea) this is a major pest of agriculture across the Americas. We used daily mortality data from a longevity experiment conducted on 487,128 male flies and 363,971 female flies (Vaupel et al. 1998). The study conditions were identical to the Million medfly study described below. The data were obtained from the DATLife database (DATLife 2017).
5. *Anastrepha obliqua* The West Indian or Antillian fruit fruit fly (Insecta:Diptera:Tephritoidea) is a major pest of mangoes. We used daily mortality data from a longevity experiment on 162,280 male and 134,807 female flies (Vaupel et al. 1998). The study conditions were identical to the Million medfly study described below. The data were obtained from the DATLife database (DATLife 2017).
6. *Anastrepha serpentina* The sapote or serpentine fruit fly (Insecta:Diptera:Tephritoidea) is a major pest in Mexico. We used daily mortality data from a longevity experiment on 172,283 male and 169,031 female flies. The study conditions were identical to Million medfly study described below. The data were obtained from the DATLife database (DATLife 2017).
7. *Diachasmimorpha longicaudata* This is a solitary parasitoid wasp (Insecta:Hymenoptera:Braconidae). It is a parasitoid of Caribbean fruit fly larvae. Daily mortality data were obtained from a longevity experiment on 13,358 male and 14,184 female wasps. The study conditions were essentially identical to the Million medfly study conducted by J. Carey and described below. Data were obtained from the DATLife database (DATLife 2017).
8. *Drosophila melanogaster* The common fruit fly (Insecta:Diptera:Drosophilidae) is probably the most widely studied laboratory organism. Life tables for this species were obtained from an early publication by Pearl and Parker (1921) on longevity experiments on long-winged and short-winged (Quintuple stock) strains. Longevity was measured for 4586 long-winged males, 5426 long-winged females, 854 short-winged males and 906 short-winged females.
9. Medfly caloric restriction experiment, *Ceratitis capitata*. The Mediterranean fruit fly (often called Medfly for short), is a species of fruit fly (Insecta:Diptera:Tephritidae) and an important fruit pest. It is native to the Mediterranean area, but has spread invasively to many parts of the world. We used data from an experiment on the effect of caloric restriction (Müller et al. 1997). Daily mortality of 200,674 males and 215,615 females, maintained in grouped cages, was observed for two caloric restriction groups: sugar and sugar plus protein. The data were obtained from the DATLife database (DATLife 2017).
10. The Million medfly experiment, *Ceratitis capitata*. The Million Medfly dataset consists of longevity data for very large cohorts of *Ceratitis capitata*. The experiment was performed in 1991 at the Moscamed medflies mass-rearing facility. The purpose of this study was to examine mortality at the extreme ages. Approximately 7200 medflies (both sexes) were maintained in each of the 167 cages. Adults were given a diet of sugar and water, ad libitum. Parts of these data were originally published in Carey et al. (1992) and Carey (1993). The dataset used here contains information on the daily numbers of age-cage-and-sex-specific deaths of the total 1,203,646 medflies (598,118 males and 605,528 females). The data were obtained from the DATLife database (DATLife 2017).

## Footnotes

1 Early failure is a concept coined in reliability engineering (Barlow and Proschan^1996^) for machines or parts that break down very soon after the onset of the use, due to some mechanical failure.
