# Supplementary material for: Variance in animal longevity: contributions of heterogeneity and stochasticity
Source: Popul Ecol. 2018 May 28;60(1):89–99. doi: 10.1007/s10144-018-0616-7 (PMC6435164; doi:10.1007/s10144-018-0616-7)
Supplement: Supplementary file 2 — Supplementary material 2 (pdf 415 KB) [file 10144_2018_616_MOESM2_ESM.pdf]

ESM-2
Variance in animal longevity: contributions of heterogeneity and stochasticity

Nienke Hartemink
Hal Caswell

January 29, 2018

Contents

1 *Caenorhabdis elegans* **2**

2 *Pediculus humanus* (Human louse) **4**

3 *Musca domestica*(Common House fly) **5**

4 *Anastrepha ludens* **7**

5 *Anastrepha obliqua* **9**

6 *Anastrepha serpentina* **11**

7 *Diachasmimorpha longicaudata* **13**

8 *Drosophila melanogaster* **15**

9 Medfly caloric restriction experiment, *Ceratitis capitata* **18**

10 The Million medfly experiment, *Ceratitis capitata* **21**

For each data set used in the analysis, we provide a short description of the species involved, the study size and the experimental design. Then, in a series of graphs, we show the distributions of age at death, first comparing the raw data, a single Weibull distribution, and the best mixture model, and then showing the distributions for the compents of the mixture model.

1 *Caenorhabdis elegans*

Caenorhabdis elegans is a free-living nematode species and an often-used model organism. Mortality data on *C. elegans* was obtained from a experiment by Chen et al. (2007). In this experiment, the longevity of a standard wild-type strain (N2) and two long-lived mutant strains (CLK-1 and DAF-2) were reported. Daily survival data on 1000 individuals of the N2 strain and 800 individuals of each of the mutant strains were available.

C. elegans: Raw data and fitted mixtures of Weibull functions

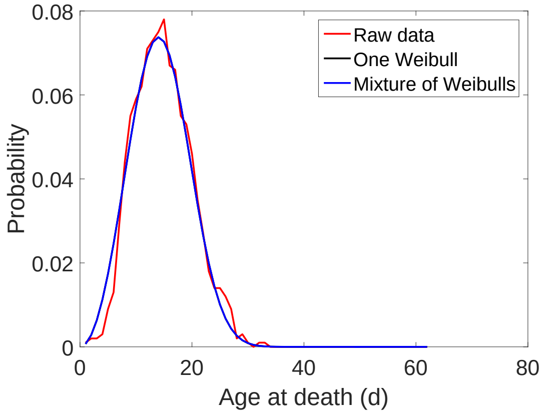

(a) N2 strain

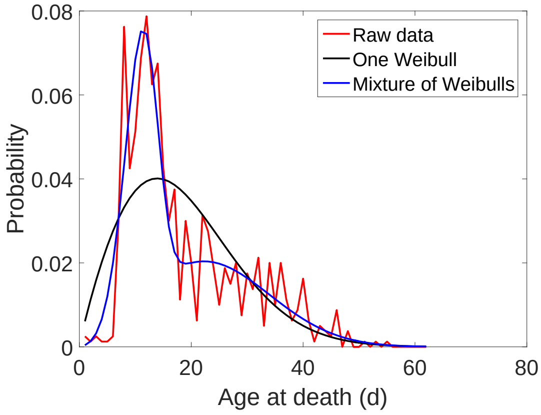

(b) CLK-1 strain

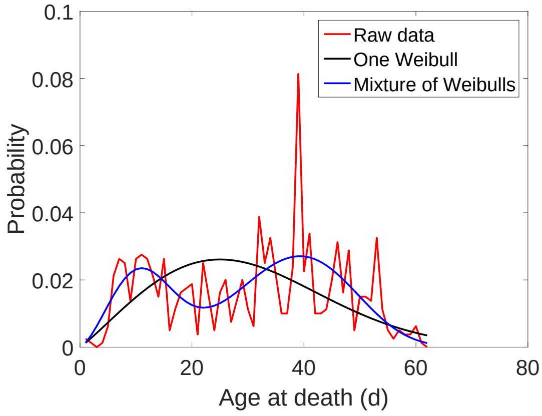

(c) DAF-2 strain

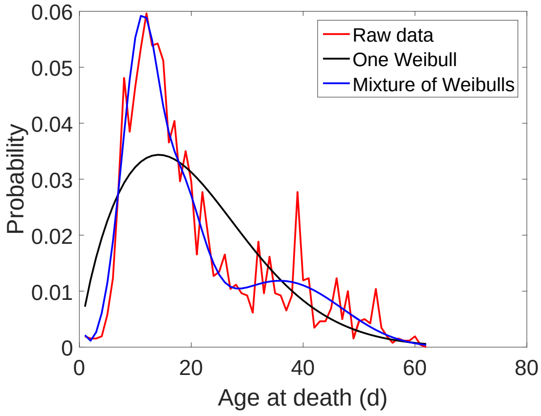

(d) All strains combined

Figure 1.1: Age-at-death of *C. elegans*: raw data (red), modelled as single Weibull function (black) and modelled as a mixture of Weibull functions(blue) for a) the N2 strain (one group), b) the CLK-1 strain (two subgroups), c) the DAF-2 strain (two subgroups) and d) all strains combined (4 subgroups).

C. elegans: Components of the mixtures

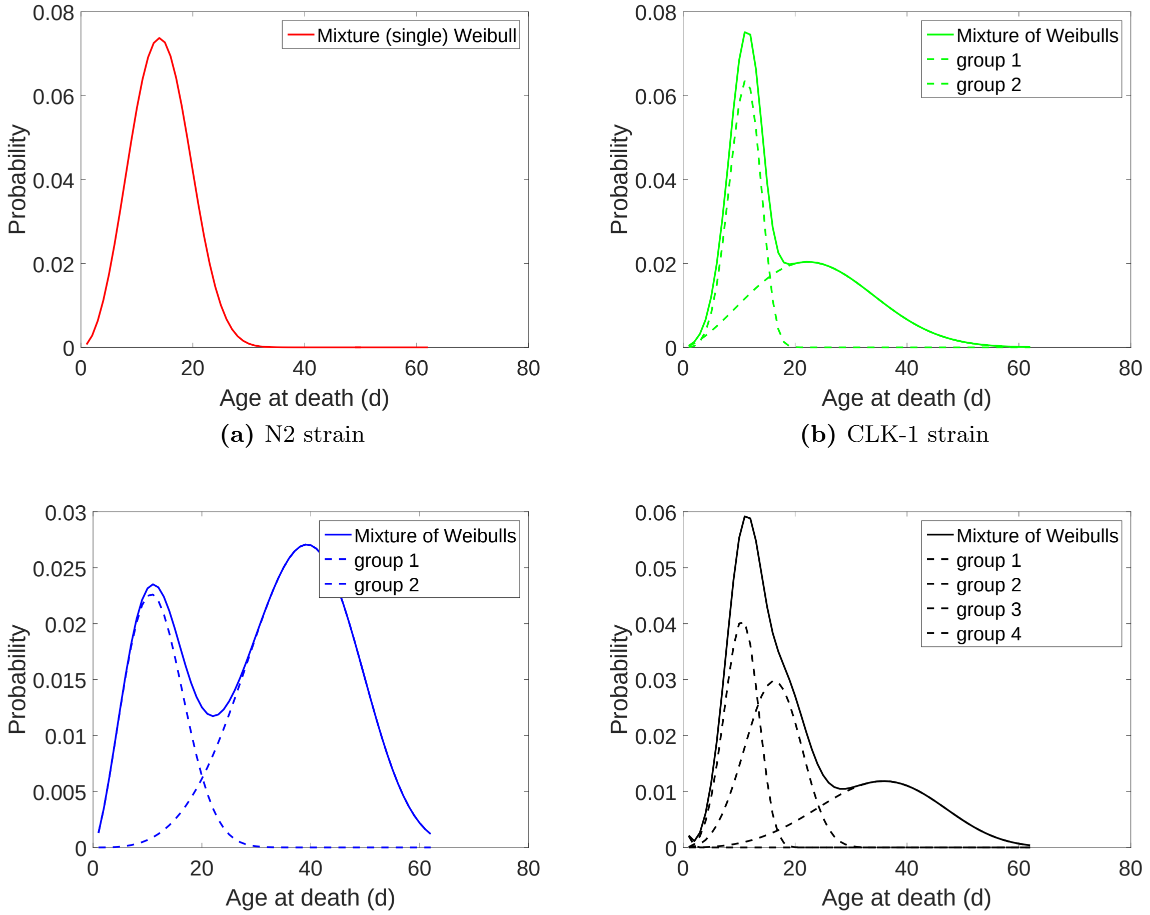

(a) N2 strain **(b)** CLK-1 strain **(c)** DAF-2 strain **(d)** All strains combined

Figure 1.2: *C. elegans*: the mixture of Weibull functions and its components for a) the N2 strain (one group), b) the CLK-1 strain (two subgroups), c) the DAF-2 strain (two subgroups) and d) all strains combined (4 subgroups).

2 *Pediculus humanus* (Human louse)

The human louse (*Pediculus humanus* L.) is a small, wingless insect. Human lice are obligate parasites of humans, that is, they normally feed exclusively in human blood, but they can be reared successfully in the lab on rabbits. The life cycle consists of the egg, three larval instars and the adult stage. Data on adult survival of head lice were obtained from a study by Evans and Smith (1952), in which 800 freshly emerged adults (400 males and 400 females) were kept in mixed colonies. The mean duration of life was 17.6 for both males and females, there was no significant difference between the two sexes.

Head louse: Raw data and fitted mixtures of Weibull functions

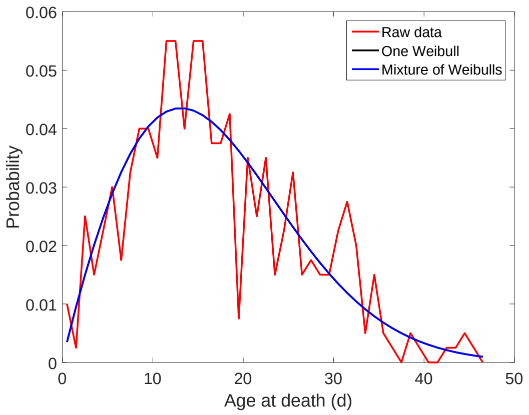

(a) Females

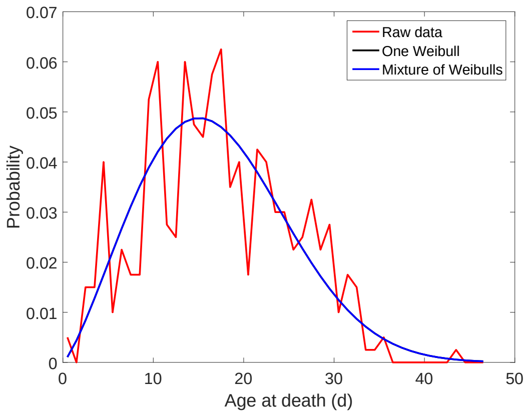

(b) Males

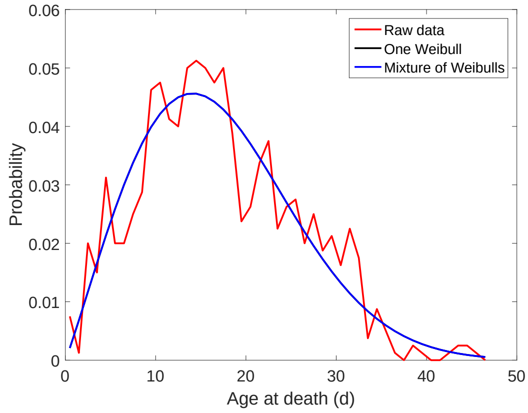

(c) Both sexes combined

Figure 2.1: Age-at-death of the Head louse: raw data (red), modelled as single Weibull function (black) and modelled as a mixture of Weibull functions (blue) for a) females (one group), b) males (one group), c) both sexes combined (one group), Since for all groups the best fit was obtained with a single Weibull function, there is no figure with curves for the subgroups for this species.

3 *Musca domestica*(Common House fly)

The common house fly (*Musca domestica* L.) is a well-known fly species (Insecta: Diptera: Muscidae). We use data on adult life span from life tables for 4,627 males and 3,875 females published in (Rockstein and Lieberman, 1959). The data originally came from 2 separate studies, but no difference in mean length of life was found between these two datasets, so we treated it as one dataset. The house flies belonged to the strain NAIDM and the flies were inbred for about 200 generations.

Housefly: Raw data and fitted mixtures of Weibull functions

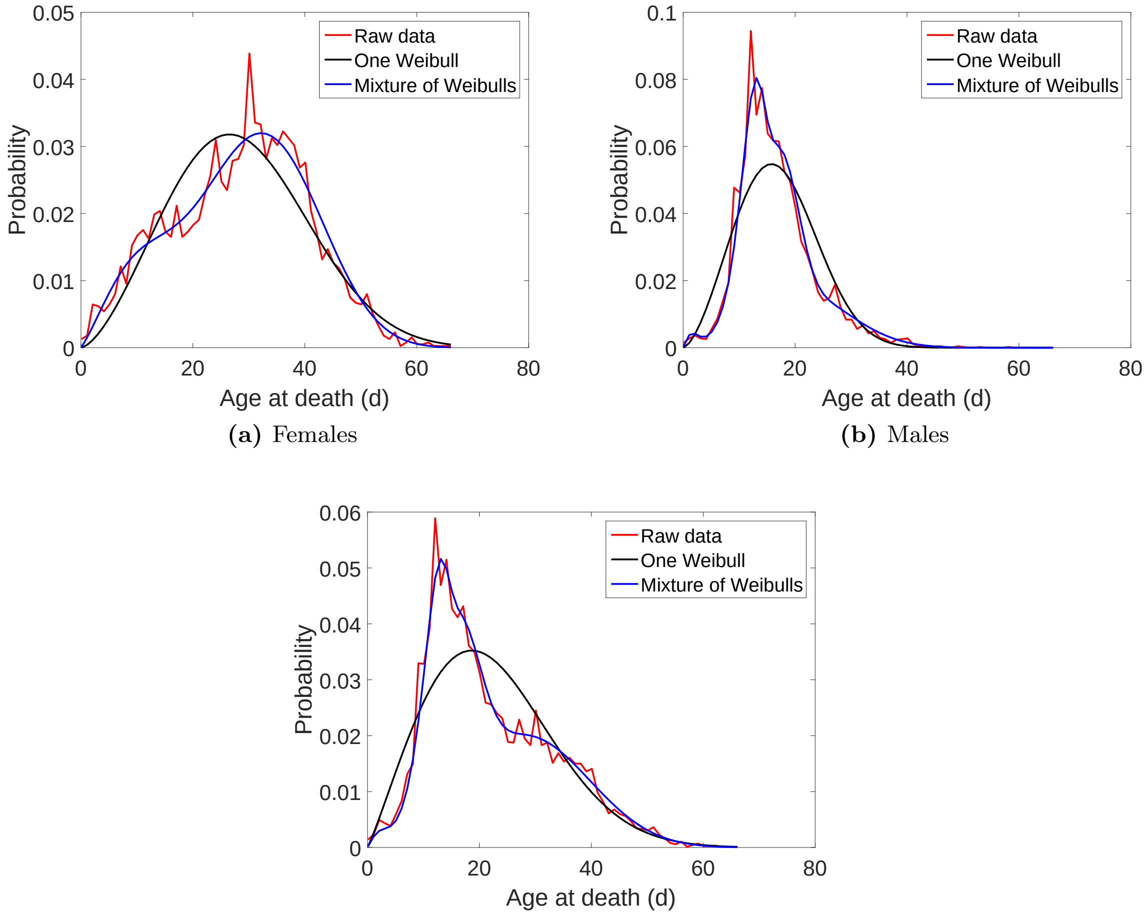

(a) Females **(b)** Males

(c) Both sexes combined

Figure 3.1: Age-at-death of the Housefly: raw data (red), modelled as single Weibull function (black) and modelled as a mixture of Weibull functions (blue) for a) females (two groups), b) males (four groups), c) both sexes combined (four groups).

Housefly: Components of the mixtures

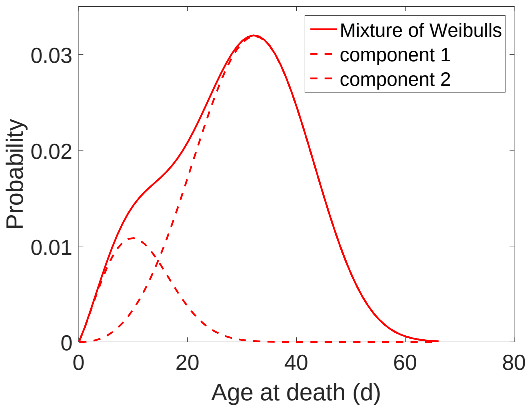

(a) Females

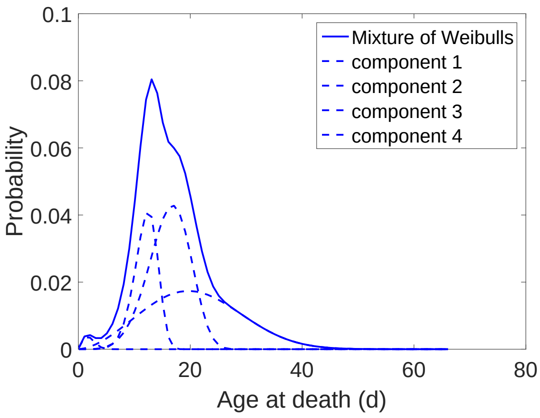

(b) Males

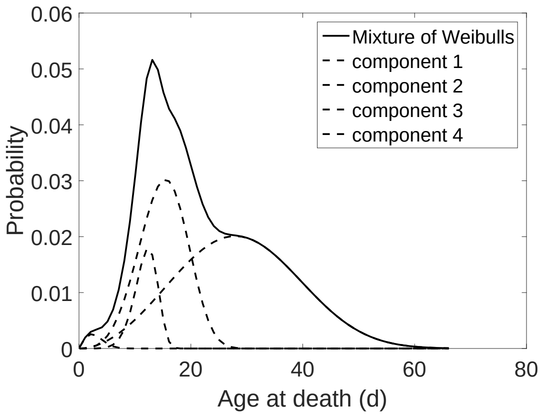

(c) Both sexes combined

Figure 3.2: Housefly: the mixture of Weibull functions and its components for a) females (two groups), b) males (four groups), c) both sexes combined (four groups).

4 *Anastrepha ludens*

Anastrepha ludens(Loew), or Mexican fruit fly, is a species of fruit fly (Insecta: Diptera: Tephritoidea); it is a major pest of agriculture across the Americas. We used daily mortality data from a longevity experiment conducted on 487,128 male flies and 363,971 female flies (Vaupel et al., 1998). The study conditions were identical to Million medfly study described below. The data were obtained from the DATLife database (DATLife, 2017).

Anastrepha ludens: Raw data and fitted mixtures of Weibull functions

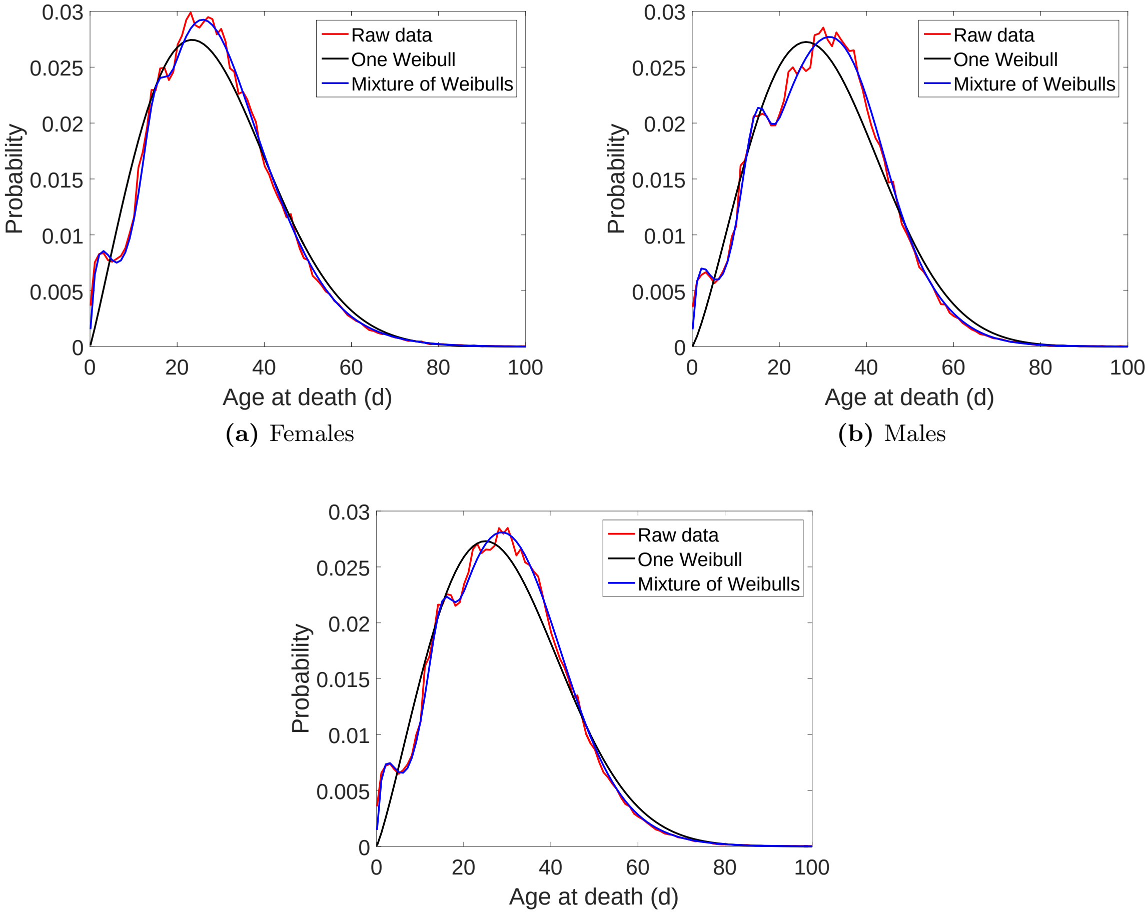

(a) Females

(b) Males

(c) Both sexes combined

Figure 4.1: Age-at-death of *Anastrepha ludens*: raw data (red), modelled as single Weibull function (black) and modelled as a mixture of Weibull functions (blue) for a) females (six groups), b) males (five groups), c) both sexes combined (five groups).

***Anastrepha ludens*: Components of the mixtures**

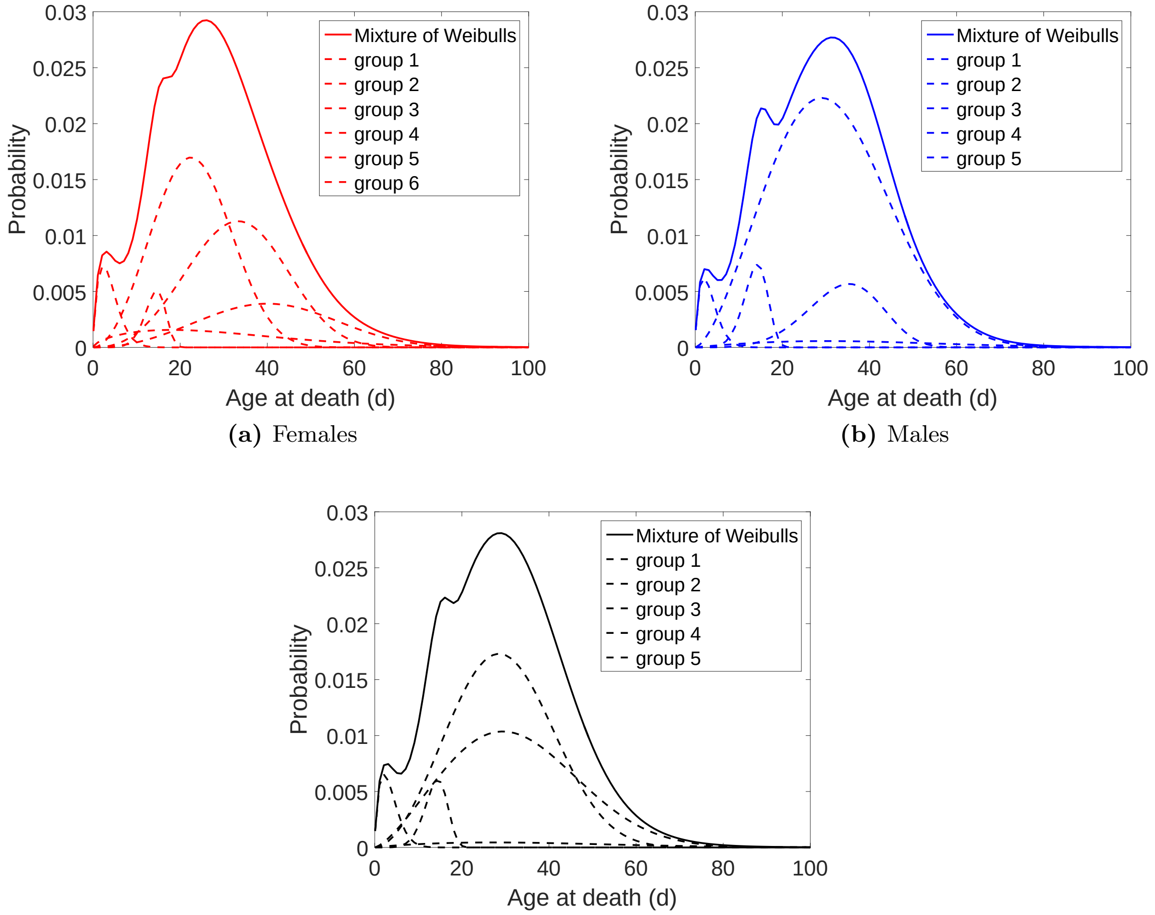

(a) Females

(b) Males

(c) Both sexes combined

Figure 4.2: *Anastrepha ludens*: the mixture of Weibull functions and its components for a) females (six groups), b) males (five groups), c) both sexes combined (five groups).

5 *Anastrepha obliqua*

Anastrepha obliqua (Macquart), the West Indian or Antillian fruit fruit fly (Insecta: Diptera: Tephritoidea) is a major pest of mangoes. We used daily mortality data from a longevity experiment on 162,280 male and 134,807 female flies (Vaupel et al., 1998). The study conditions were identical to Million medfly study described below. The data were obtained from the DATLife database (DATLife, 2017).

Anastrepha obliqua: Raw data and fitted mixtures of Weibull functions

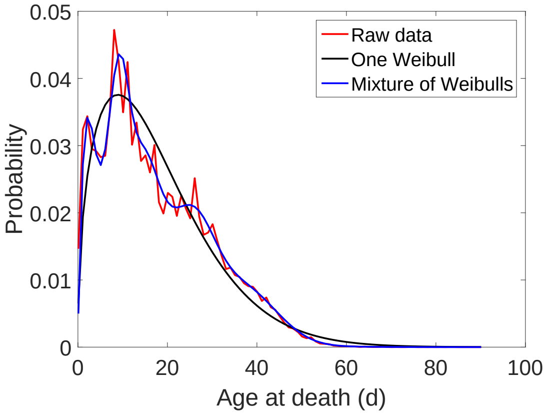

(a) Females

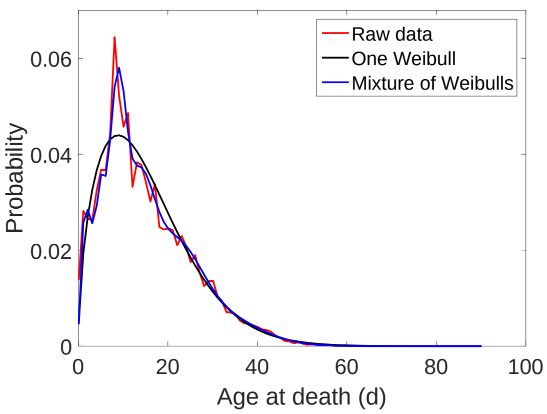

(b) Males

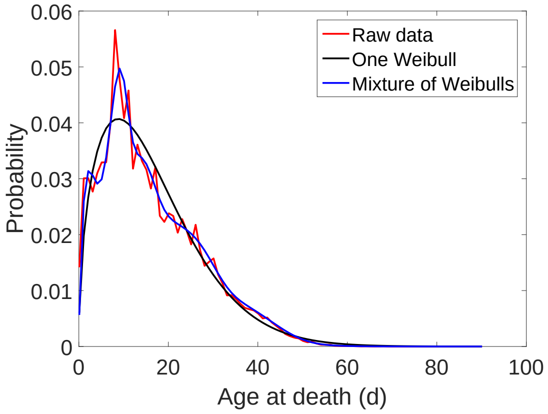

(c) Both sexes combined

Figure 5.1: Age-at-death of *Anastrepha obliqua*: raw data (red), modelled as single Weibull function (black) and modelled as a mixture of Weibull functions (blue) for a) females (six groups), b) males (six groups), c) both sexes combined (six groups).

Anastrepha obliqua: Components of the mixtures

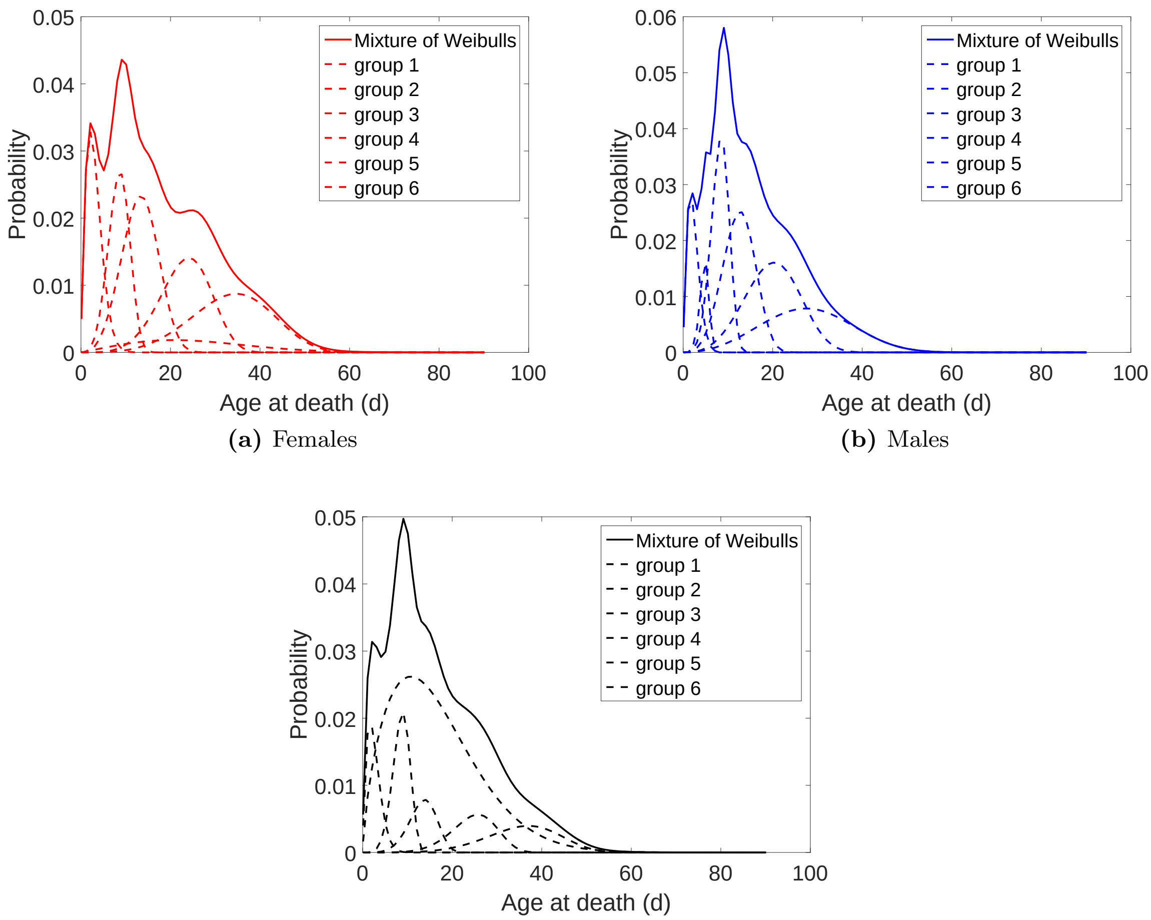

(a) Females

(b) Males

(c) Both sexes combined

Figure 5.2: *Anastrepha obliqua*: the mixture of Weibull functions and its components for a) females (six groups), b) males (six groups), c) both sexes combined (six groups).

6 *Anastrepha serpentina*

Anastrepha serpentina(Wiedemann), the sapote or serpentine fruit fly (Insecta: Diptera: Tephritoidea) is a major pest in Mexico. We used daily mortality data from a longevity experiment on 172,283 male and 169,031 female flies. The study conditions were identical to Million medfly study described below. The data were obtained from the DATLife database (DATLife, 2017).

Anastrepha serpentina: Raw data and fitted mixtures of Weibull functions

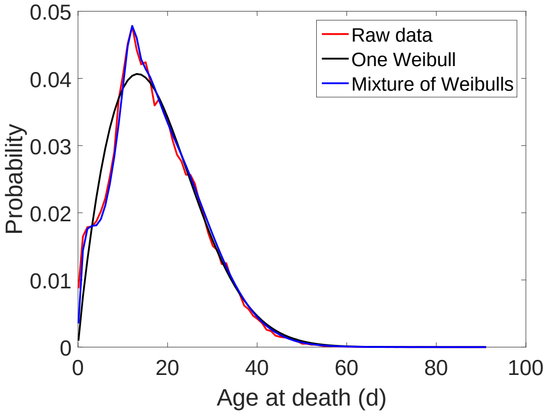

(a) Females

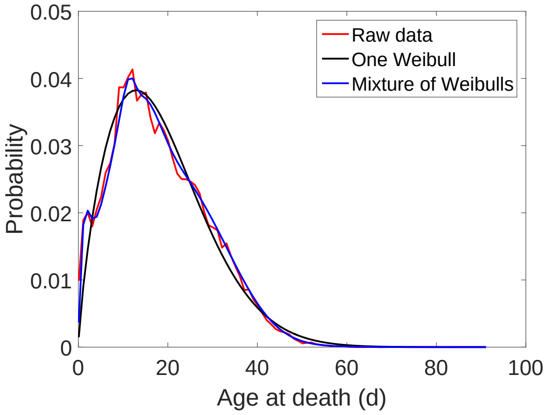

(b) Males

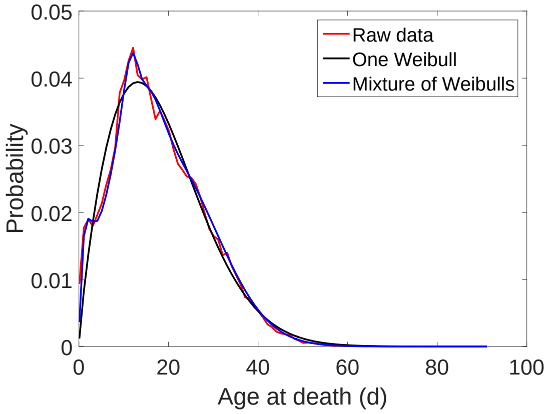

(c) Both sexes combined

Figure 6.1: Age-at-death of *Anastrepha serpentina*: raw data (red), modelled as single Weibull function (black) and modelled as a mixture of Weibull functions (blue) for a) females (five groups), b) males (five groups), c) both sexes combined (five groups).

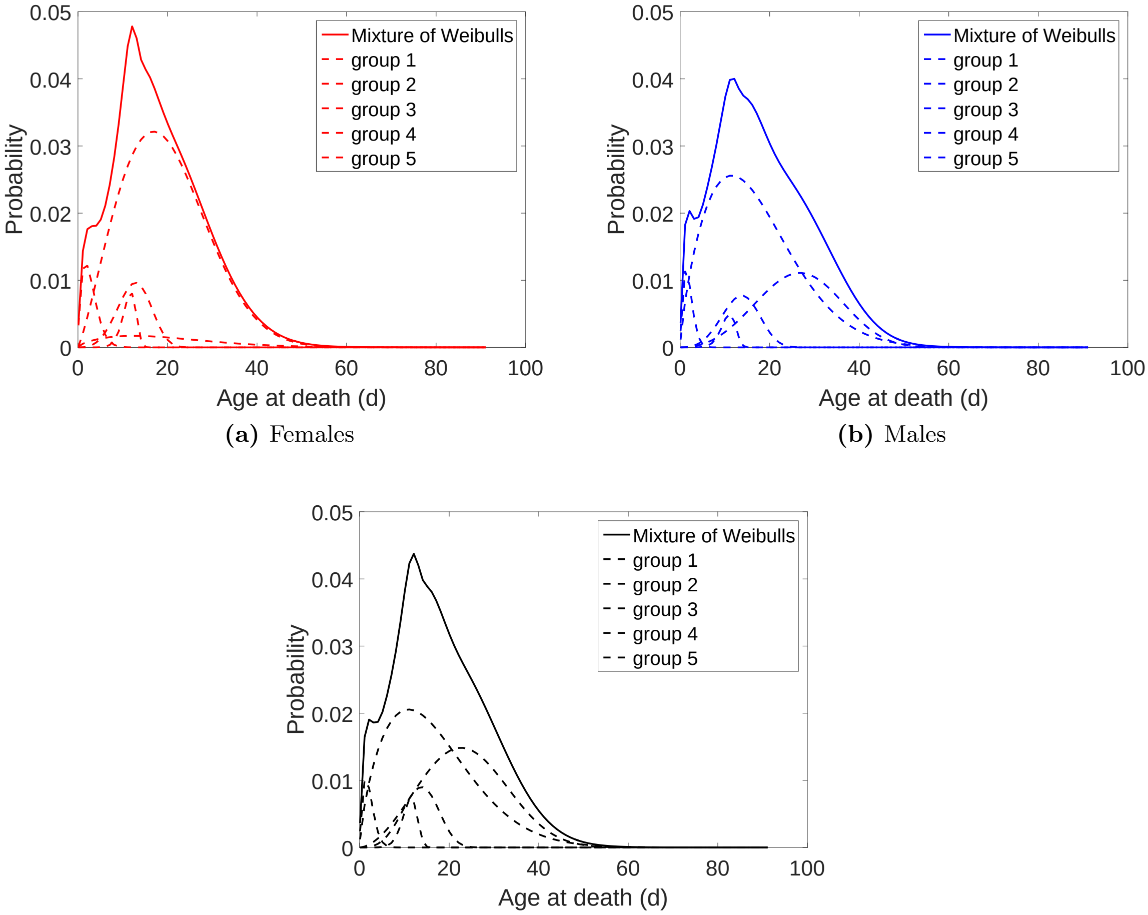

(a) Females

(b) Males

(c) Both sexes combined

Figure 6.2: *Anastrepha serpentina*: the mixture of Weibull functions and its components for a) females (five groups), b) males (five groups), c) both sexes combined (five groups).

7 *Diachasmimorpha longicaudata*

Diachasmimorpha longicaudata (Ashmead) is a solitary parasitoid wasp (Insecta: Hymenoptera: Braconidae). It is a parasitoid of Caribbean fruit fly larvae. Daily mortality data were obtained from a longevity experiment on 13,358 male and 14,184 female wasps. The study conditions were essentially identical to the Million medfly study conducted by J. Carey and described below. Data were obtained from the DATLife database (DATLife, 2017).

D. longicaudata: Raw data and fitted mixtures of Weibull functions

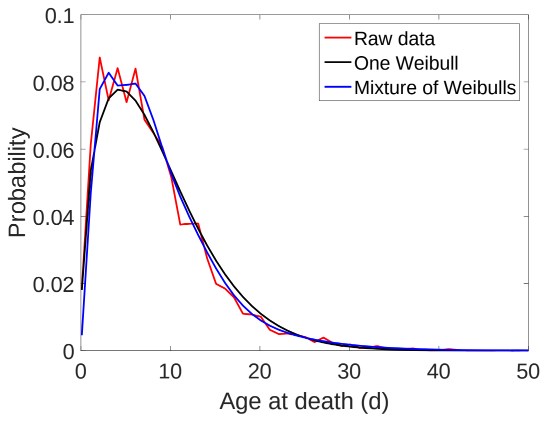

(a) Females

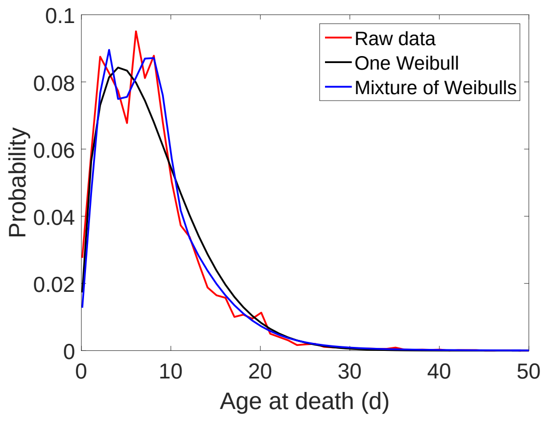

(b) Males

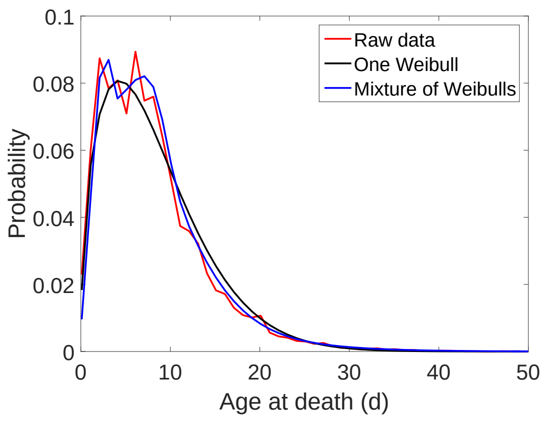

(c) Both sexes combined

Figure 7.1: Age-at-death of *D. longicaudata*: raw data (red), modelled as single Weibull function (black) and modelled as a mixture of Weibull functions (blue) for a) females (four groups), b) males (four groups), c) both sexes combined (four groups).

D. longicaudata: Components of the mixtures

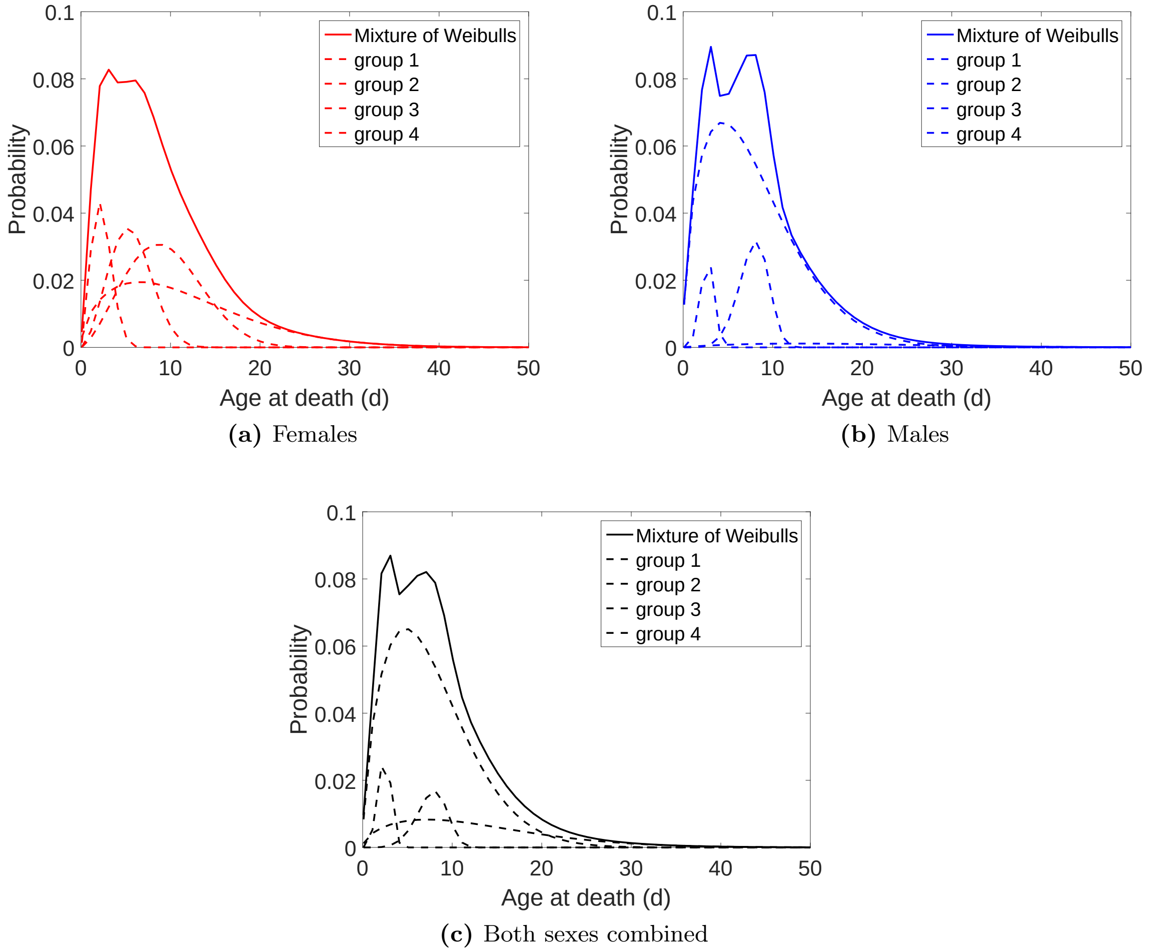

Figure 7.2: *D. longicaudata*: the mixture of Weibull functions and its components for a) females (four groups), b) males (four groups), c) both sexes combined (four groups).

8 *Drosophila melanogaster*

Drosophila melanogaster, known generally as the common fruit fly or vinegar fly, is a species of fly (Insecta: Diptera: Drosophilidae). Life tables for this species were obtained from a publication by Pearl and Parker (1921) on longevity experiments on long-winged and short-winged (Quintuple stock) strains. Longevity was measured for 4,586 long-winged males, 5,426 long-winged females, 854 short-winged males and 906 short-winged females.

Drosophila melanogaster: Raw data and fitted mixtures of Weibull functions

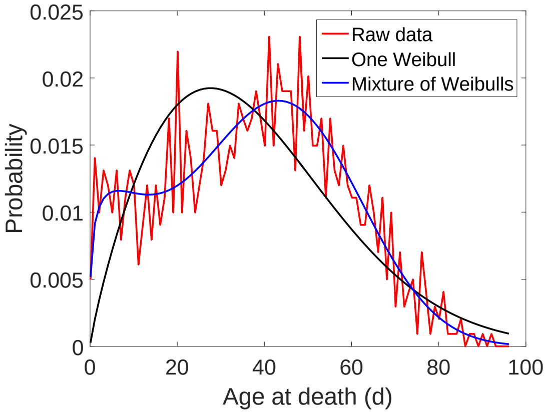

(a) Long-winged females

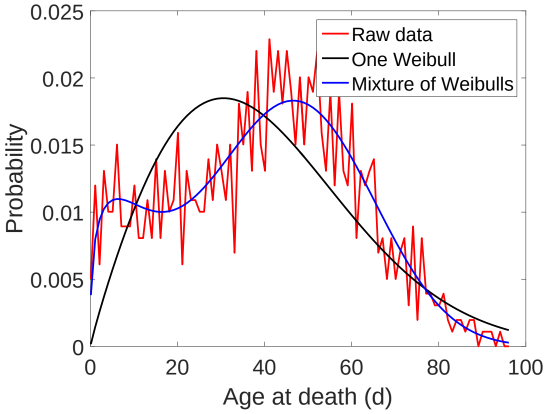

(b) Long-winged males

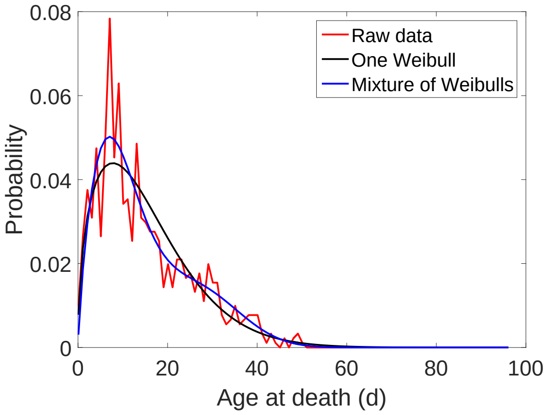

(c) Short-winged females

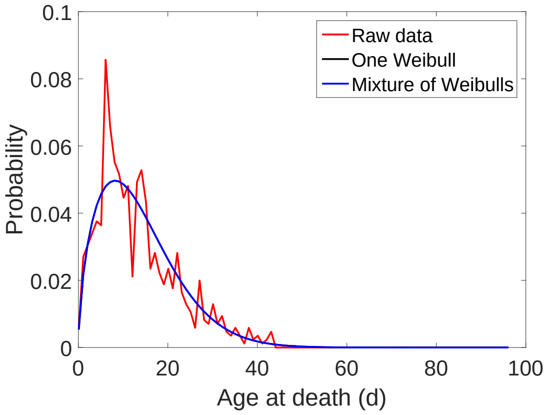

(d) Short-winged males

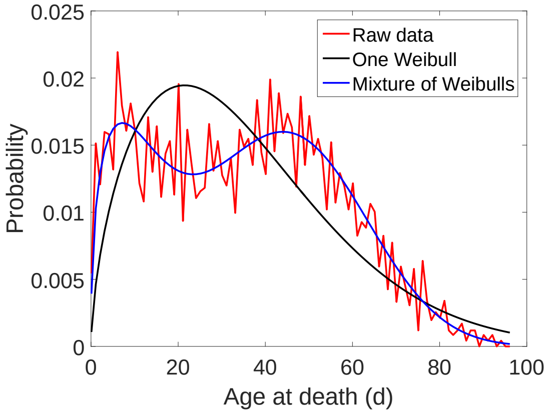

(e) All groups combined

Figure 8.1: Age-at-death of *D. melanogaster*: raw data (red), modelled as single Weibull function (black) and modelled as a mixture of Weibull functions (blue) for a) long-winged females (two groups), b) long-winged males (two groups), c) short-winged females (two groups), d) short-winged males (one group) and e) all groups combined (two groups).

Drosophila melanogaster: Components of the mixtures

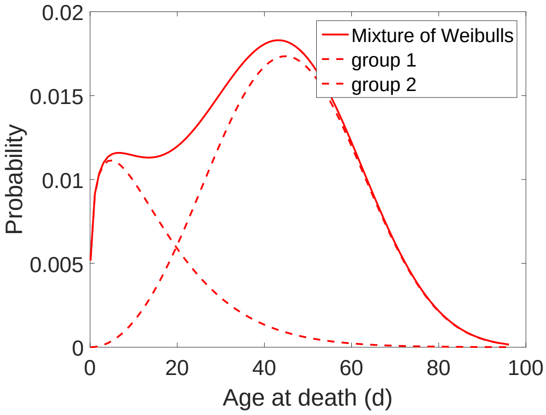

(a) Long-winged females

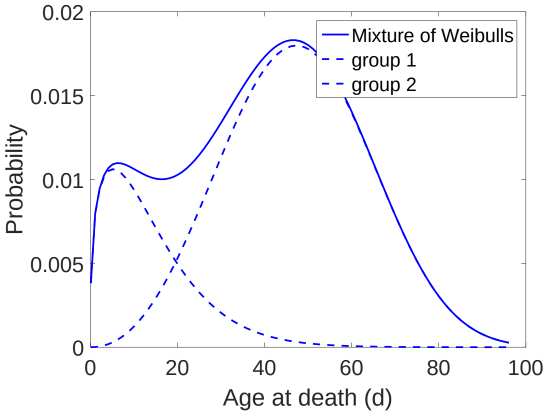

(b) Long-winged males

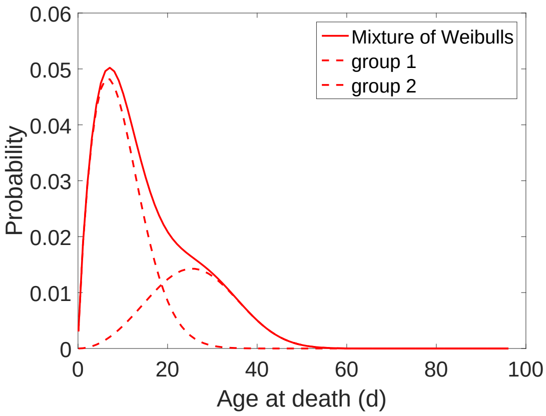

(c) Short-winged females

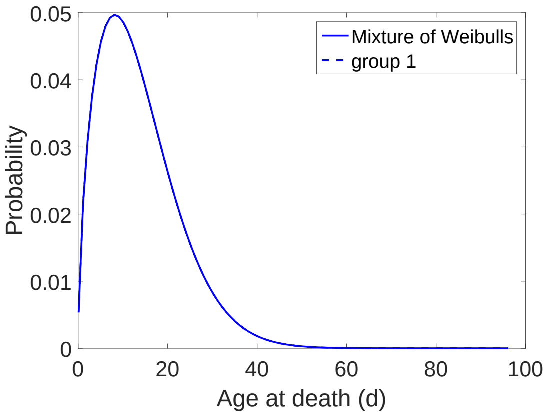

(d) Short-winged males

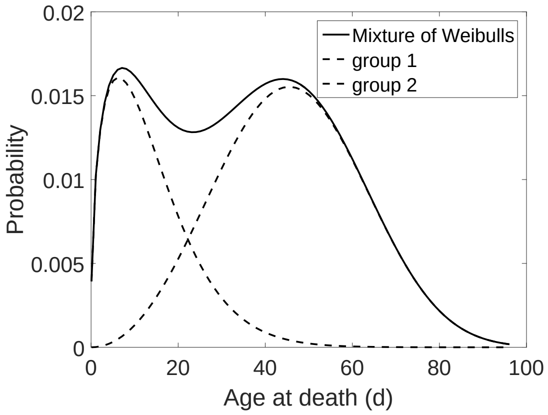

(e) All groups combined

Figure 8.2: *D. melanogaster*: the mixture of Weibull functions and its components for a) long-winged females (two groups), b) long-winged males (two groups), c) short-winged females (two groups), d) short-winged males (one group) and e) all groups combined (two groups).

9 Medfly caloric restriction experiment, *Ceratitis capitata*

Ceratitis capitata (Wiedemann), the Mediterranean fruit fly (often called medfly for short), is a species of fruit fly (Insecta: Diptera: Tephritidae) and an important fruit pest. It is native to the Mediterranean area, but has spread invasively to many parts of the world. We used data from an experiment on the effect of caloric restriction (Müller et al., 1997). Daily mortality of 200,674 males and 215,615 females, maintained in grouped cages, was observed for two caloric restriction groups: sugar and sugar plus protein. The data were obtained from the DATLife database (DATLife, 2017).

Ceratitis capitata: Raw data and fitted mixtures of Weibull functions

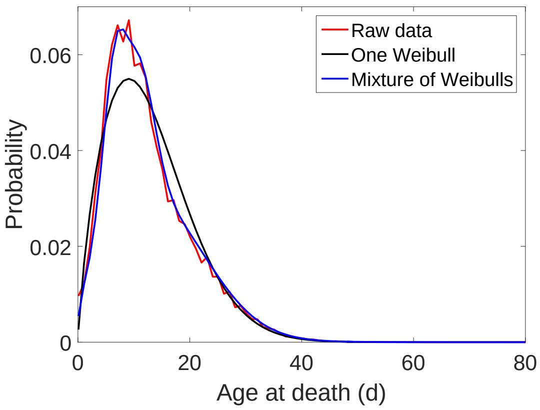

(a) Sugar-fed medfly females

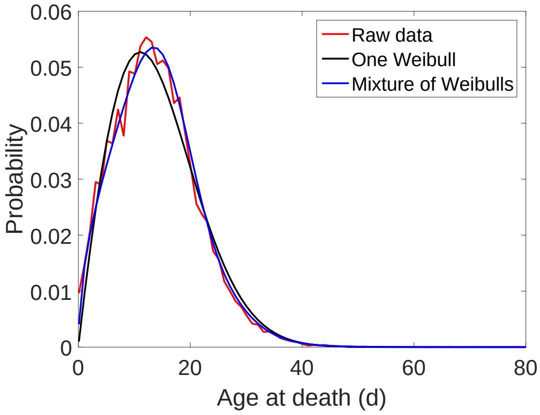

(b) Sugar-fed medfly males

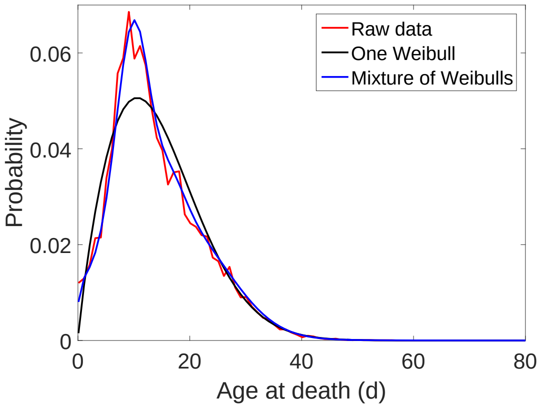

(c) Sugar and protein-fed medfly females

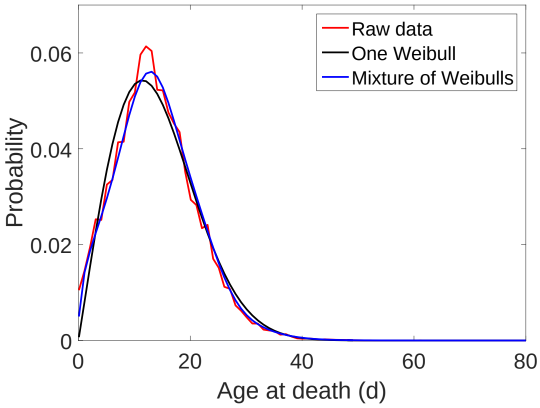

(d) Sugar and protein-fed males

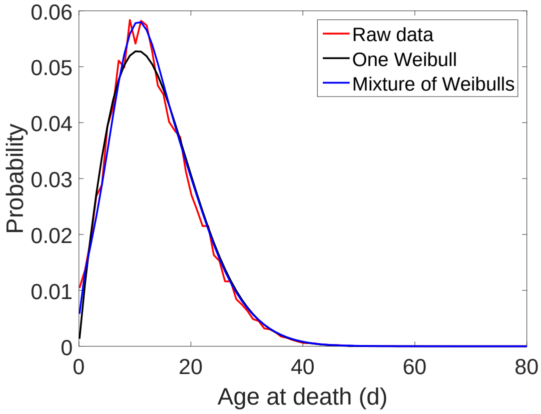

(e) All groups combined

Figure 9.1: Age-at-death of *Ceratitis capitata*: raw data (red), modelled as single Weibull function (black) and modelled as a mixture of Weibull functions (blue) for a) sugar-fed females (two groups), b) sugar-fed males (four groups), c) sugar and protein-fed females (four groups), d) sugar and protein-fed males (four groups) and e) all groups combined (four groups).

Ceratitis capitata: Components of the mixtures

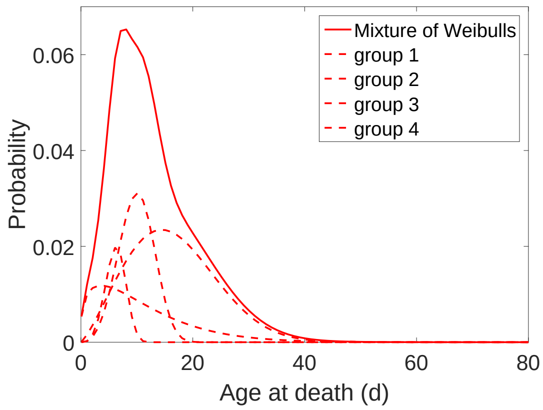

(a) Sugar-fed females

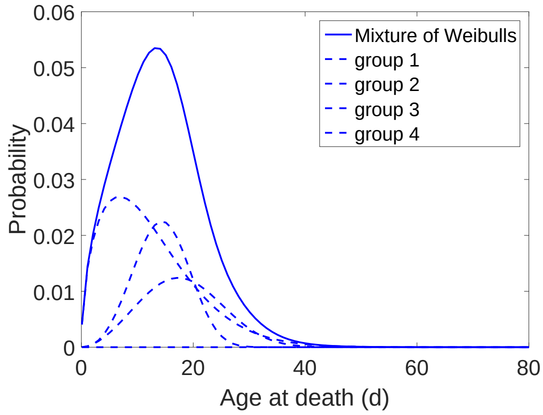

(b) Sugar-fed males

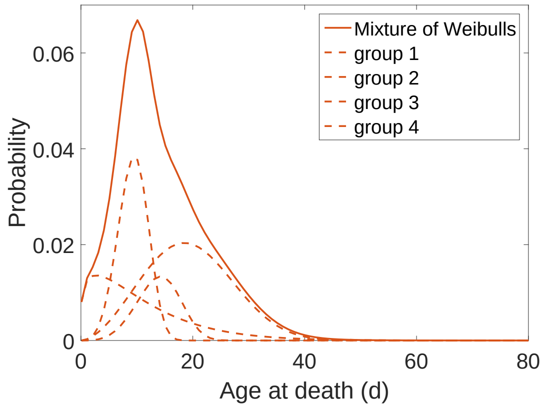

(c) Sugar and protein-fed females

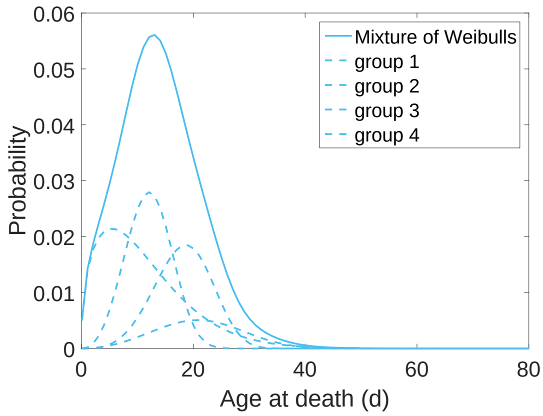

(d) Sugar and protein-fed males

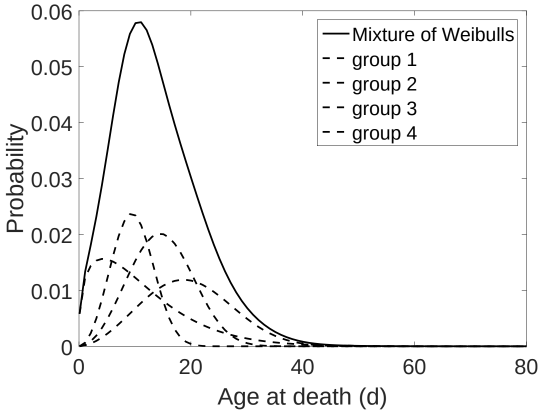

(e) All groups combined

Figure 9.2: *Ceratitis capitata*: the mixture of Weibull functions and its components for a) sugar-fed females (two groups), b) sugar-fed males (four groups), c) sugar and protein-fed females (four groups), d) sugar and protein-fed males (four groups) and e) all groups combined (four groups).

10 The Million medfly experiment, *Ceratitis capitata*

Ceratitis capitata (Wiedemann), the Mediterranean fruit fly (often called medfly for short), is a species of fruit fly (Insecta: Diptera: Tephritidae) is an important fruit pest. It is native to the Mediterranean area, but has spread invasively to many parts of the world. The famous Million Medfly dataset consists of longevity data for very large cohorts of *Ceratitis capitata*. The experiment was performed in 1991 at the Moscamed medflies mass-rearing facility. The purpose of this study was to examine mortality at the extreme ages. Approximately 7200 medflies (both sexes) were maintained in each of the 167 cages. Adults were given a diet of sugar and water, ad libitum. Parts of these data were originally published in (Carey et al., 1992; Carey, 1993). The dataset used here contains information on the daily numbers of age-cage-and-sex-specific deaths of the in total 1,203,646 medflies (598,118 males and 605,528 females). The data were obtained from the DATLife database (DATLife, 2017).

Ceratitis capitata in Million medfly experiment: Raw data and fitted mixtures of Weibull functions

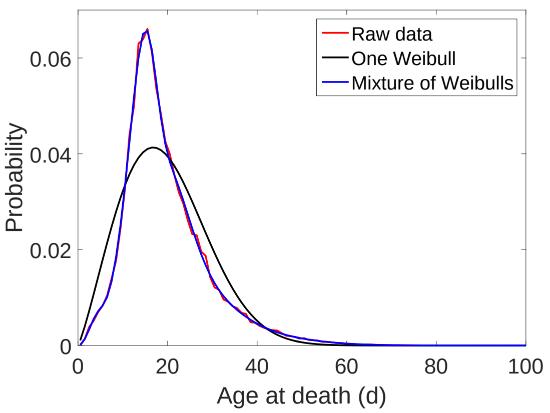

(a) Females

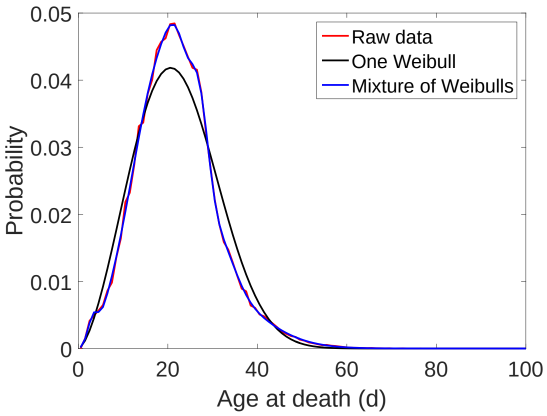

(b) Males

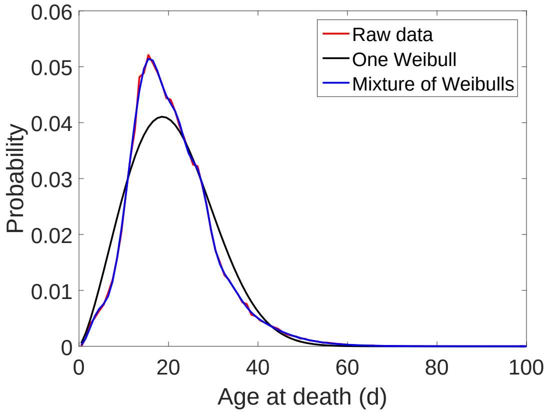

(c) Both sexes combined

Figure 10.1: Age-at-death of *Ceratitis capitata*: raw data (red), modelled as single Weibull function (black) and modelled as a mixture of Weibull functions (blue) for a) females (six groups), b) males (eight groups), c) both sexes combined (eight groups).

Ceratitis capitata in Million medfly experiment: Components of the mixtures

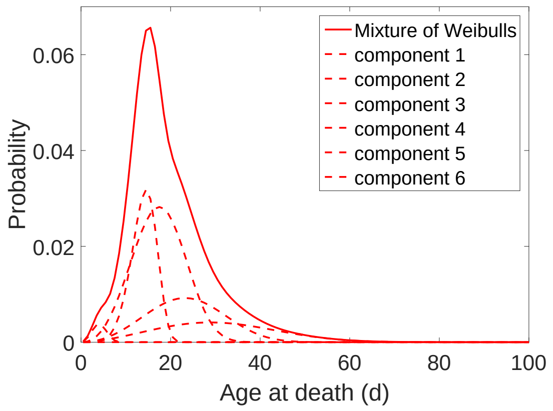

(a) Females

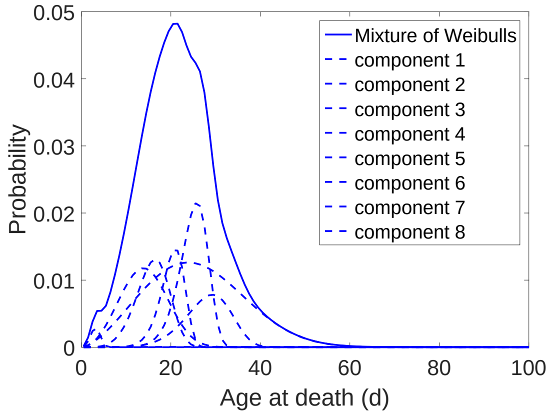

(b) Males

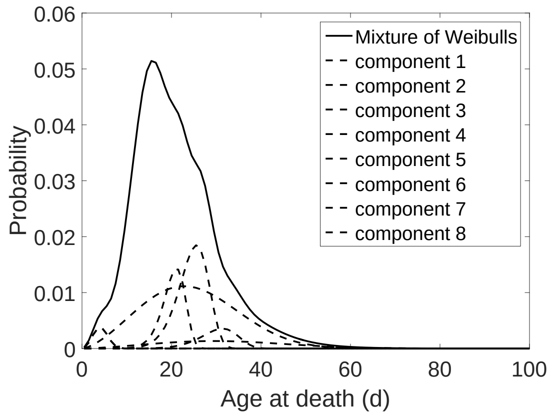

(c) Both sexes combined

Figure 10.2: *Ceratitis capitata*: the mixture of Weibull functions and its components for a) females (six groups), b) males (eight groups), c) both sexes combined (eight groups).

References

Carey, J. R. 1993. Applied demography for biologists: with special emphasis on insects. Oxford University Press, Oxford, United Kingdom.

Carey, J. R., P. Liedo, D. Orozco, and J. W. Vaupel. 1992. Slowing of mortality rates at older ages in large medfly cohorts. Science **258**:457–91.

Chen, J., D. Senturk, J.-L. Wang, H.-G. Müller, J. R. Carey, H. Caswell, and E. P. Caswell-Chen. 2007. A demographic analysis of the fitness cost of extended longevity in *Caenorhabditis elegans*. The Journals of Gerontology Series A: Biological Sciences and Medical Sciences **62**:126–135.

DATLife, 2017. Database: Demography of Aging Across the Tree of Life. Max Planck Institute for Demographic Research. URL `https://datlife.org`.

Evans, F. C., and F. E. Smith. 1952. The intrinsic rate of natural increase for the human louse, *Pediculus humanus* L. American Naturalist **86**:299–310.

Müller, H.-G., J.-L. Wang, W. B. Capra, P. Liedo, and J. R. Carey. 1997. Early mortality surge in protein-deprived females causes reversal of sex differential of life expectancy in Mediterranean fruit flies. Proceedings of the National Academy of Sciences **94**:2762–2765.

Pearl, R., and S. L. Parker. 1921. Experimental studies on the duration of life. I. Introductory discussion of the duration of life in *Drosophila*. American Naturalist pages 481–509.

Rockstein, M., and H. M. Lieberman. 1959. A life table for the common house fly, *Musca domestica*. Gerontology **3**:23–36.

Vaupel, J. W., J. R. Carey, K. Christensen, T. E. Johnson, A. I. Yashin, N. V. Holm, I. A. Iachine, V. Kannisto, A. A. Khazaeli, P. Liedo, V. D. Longo, Y. Zeng, K. G. Manton, and J. W. Curtsinger. 1998. Biodemographic trajectories of longevity. Science **280**:855–860.
